# Enhancing immunotherapy through PD‐L1 upregulation: the promising combination of anti‐PD‐L1 plus mTOR inhibitors

**DOI:** 10.1002/1878-0261.13699

**Published:** 2024-09-11

**Authors:** Anna Hernández‐Prat, Alejo Rodriguez‐Vida, Laura Cardona, Mengjuan Qin, Oriol Arpí‐Llucià, Luis Soria‐Jiménez, Sílvia Menendez, Fabricio Gerel Quimis, Miguel Galindo, Edurne Arriola, Marta Salido, Nuria Juanpere‐Rodero, Federico Rojo, Aura Muntasell, Joan Albanell, Ana Rovira, Joaquim Bellmunt

**Affiliations:** ^1^ Cancer Research Programe IMIM (Hospital del Mar Research Institute) Barcelona Spain; ^2^ Medical Oncology Department Hospital del Mar‐CIBERONC Barcelona Spain; ^3^ Pathology Department Hospital del Mar‐CIBERONC Barcelona Spain; ^4^ Pathology Department IIS Fundación Jimenez Diaz‐CIBERONC Madrid Spain; ^5^ Immunity and Infection Group IMIM (Hospital del Mar Research Institute)‐CIBERONC Barcelona Spain; ^6^ Universitat Autònoma de Barcelona Spain; ^7^ Dana Farber Medical Institute Harvard Medical School Boston MA USA

**Keywords:** bladder cancer, HLA‐I, mTOR, PD‐L1, tumor microenvironment

## Abstract

Immune checkpoint inhibitors (ICIs) targeting the programmed cell death protein 1 (PD‐1)/programmed cell death 1 ligand 1 (PD‐L1) pathway have transformed urothelial cancer (UC) therapy. The correlation between PD‐L1 expression and ICI effectiveness is uncertain, leaving the role of PD‐L1 as a predictive marker for ICI efficacy unclear. Among several ways to enhance the efficacy of ICI, trials are exploring combining ICIs with serine/threonine‐protein kinase mTOR (mTOR) inhibitors in different tumor types. The potential interaction between mTOR inhibitors and PD‐L1 expression in UC has not been well characterized. In our study, we investigated how phosphoinositide 3‐kinase (PI3K)/AKT/mTOR pathway inhibitors (TAK‐228, everolimus and TAK‐117) affect PD‐L1 expression and function in preclinical bladder cancer cell models. TAK‐228 increased cell surface levels of glycosylated PD‐L1 in all but one of the seven cell lines, regardless of baseline levels. TAK‐228 promoted the secretion of epidermal growth factor (EGF) and interferon‐β (IFNβ), both linked to PD‐L1 protein induction. Blocking EGF and IFNβ receptors reversed the TAK‐228‐induced PD‐L1 increase. Additionally, TAK‐228 enhanced IFN‐γ‐induced PD‐L1 expression and intracellular HLA‐I levels in some cells. TAK‐228‐treated bladder cancer cells exhibited resistance to the cytotoxic effects of peripheral blood mononuclear cells (PBMCs) and cluster of differentiation 8 (CD8)+ T cells. The addition of an anti‐PD‐L1 antibody diminished this resistance in T24 cells. Increased expression of PD‐L1 under TAK‐228 exposure was confirmed in patient‐derived explants (PDEs) treated *ex vivo*. These preclinical findings suggest that mTOR inhibition with TAK‐228 can increase PD‐L1 levels, potentially impacting the specific immune response against UC cells. This highlights the rationale for exploring the combination of mTOR inhibitors with ICIs in patients with advanced UC.

## Graphical abstract

mTOR inhibition with TAK‐228 can increase PD‐L1 levels, potentially impacting the specific immune response against bladder cancer cell lines.

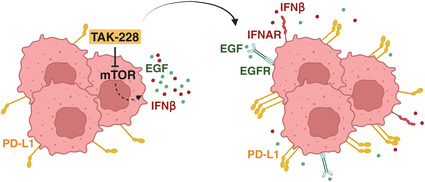

**Abbreviations**
BC: bladder cancer
CD8: cluster of differentiation 8
EGF: epidermal growth factor
EGFR: epidermal growth factor receptor
GSK3β: glycogen synthase kinase 3 beta
HIF‐1α (HIF1A): hypoxia‐inducible factor 1‐alpha
ICI: immune checkpoint inhibitors
IFNAR2: interferon receptor chain 2
IFNβ: interferon‐beta
IFNγ: interferon‐gamma
IL‐6: interleukin‐6
mTOR: mammalian target of rapamycin
PBMC: peripheral blood mononuclear cells
PD‐1: programmed cell death 1
PD‐L1: programmed cell death‐ligand 1
S6: ribosomal protein S6
UC: urothelial cancer
α‐IFNAR2: anti‐interferon alpha receptor 2

## 1 Introduction

Immune checkpoint inhibitors (ICI) have transformed the treatment across various cancers, including urothelial cancer (UC). PD‐1/PD‐L1 inhibitors have yielded improved survival and maintained durable responses in a limited proportion of patients, to an extent never seen before in advanced UC. The PD‐1 inhibitor pembrolizumab showed for the first time in history an improvement in overall survival compared to standard chemotherapy in a phase III randomized trial in patients with metastatic UC progressing to platinum‐based chemotherapy [1]. Pembrolizumab has thus become the second‐line standard‐of‐care treatment for metastatic UC. More recently, maintenance avelumab (PD‐L1 inhibitor) has showed improved overall survival compared to best supportive care in patients with advanced UC with disease response or stabilization after first‐line platinum‐based chemotherapy [2]. Nivolumab (PD‐1 inhibitor) enhances disease‐free survival in patients with muscle‐invasive UC [3]. Despite these advances, the overall objective response rates (ORR) of these ICI varies only around 20–23% indicating that a great majority of patients might not benefit from the use of ICI treatment. Similarly, only half of the responders have maintained durable remissions with most of the patients ultimately experiencing disease progression. Consequently, innovative strategies for improving the efficacy of ICI in metastatic UC are urgently needed.

Aside from adoptive T cell therapy, cancer vaccines, and monoclonal antibodies targeting new agonist or antagonist immune checkpoints, most efforts aiming to improve cancer immunotherapy are focusing on immunotherapy‐based combinations [4]. Different strategies combining ICI with other immunotherapy agents, chemotherapy, radiotherapy, and targeted agents are currently being studied. However, not all these combinations have been built on a robust scientific background rationale exception made of the combination of pembrolizumab/enfortumab vedotin (EV/P) that is now FDA approved based on the high rate of responses and durability seen in unfit patients in first line [5]. Pending final results of the phase III trial comparing EV/P versus standard of care, (with a recent press release on Sept 22nd reporting positive results) additional research on improved combinations is needed.

In order to maximize the benefit of ICI‐based combinations, active research should focus in developing a strong preclinical rational to support each combination based on predictive biomarkers according to the molecular profile of the disease. Preclinical studies with mouse or human cancer cells *in vitro* and *in vivo* have demonstrated anti‐tumoral efficacy of combining mTOR inhibitors with ICI such as PD‐1/PD‐L1 inhibitors and others [6, 7]. Clinical trials are investigating the combination of PI3K or AKT inhibitors in combination with PD‐1/PD‐L1 inhibitors in solid tumors [8]. In UC, anti‐PD‐1/PD‐L1 therapy has been evaluated in combination with different targeted therapies such as FGFR inhibitors, PARP inhibitors, and EHZ2 inhibitor among others [9, 10]. The BISCAY phase 2 trial (NCT02546661) investigated the role of combining the anti‐PD‐L1 therapy durvalumab, with several targeted therapies including an mTOR inhibitor in metastatic UC [11] with no proven significant benefit with any of the combinations. Despite some early negative trials, the possible crosstalk/synergy between the mTOR and the PD‐1/PD‐L1 pathways remains still widely unexplored. Bladder cancer (BC) has some frequent mTOR alterations, with the most common mutations occurring in the TSC1 and PIK3CA genes, as well as copy number alterations affecting the PIK3CA gene. Given its prevalence as one of the most common types of UC, we focus our study in this type of cancer.

In this article, we aimed to characterize the effects of three inhibitors of the PI3K/AKT/mTOR pathway (TAK‐228, everolimus and TAK‐117) on the regulation and function of the PD‐1/PD‐L1 pathway in BC models and to explore whether the effects of the mTOR pathway inhibition in cancer cells may affect the anti‐tumor immune responses.

## 2 Materials and methods

### 2.1 Cell lines and cell culture conditions

Human bladder cancer cells, including T24 (CVCL_0554), HT‐1197 (CVCL_1291), TCCSUP (CVCL_1738), UM‐UC‐3 (CVCL_1783), J82 (CVCL_0359), RT4 (CVCL_0036) (obtained from American Type Culture Collection, ATCC, Manassas, VA, USA), and CAL‐29 (CVCL_1808) (obtained from DSMZ‐German Collection of Microorganisms and Cell Cultures), were grown in Minimum Essential Medium supplemented with l‐glutamine (2 mm·L^−1^), penicillin/streptomycin (100 U/100 μg·mL^−1^) (Live Technologies, Thermo Fisher Scientific, Waltham, MA, USA) and 10% fetal bovine serum (FBS) (Sigma‐Aldrich, St. Louis, MO, USA). The cells were maintained at 37 °C under a 5% CO_2_ humidified atmosphere. The number of passages conducted for the described experiments was 15 or fewer. Cell authentication was performed using short‐tandem repeat DNA profiling recommended by ATCC experts. The detection of *Mycoplasma* contamination was carried out at the cell culture core facility of our institution.

### 2.2 Generation of T24‐GFP+‐Luc+ and CAL‐29‐GFP+‐Luc+ cells

T24‐GFP+‐Luc+ and CAL‐29‐GFP+‐Luc+ were generated from parental cell lines T24 and CAL‐29, respectively, by stable lentiviral transduction. The plasmid coding for GFP and Luciferase, pHIV‐Luc‐ZsGreen (#39196; Addgene, Watertown, MA, USA), was packaged into lentivirus by transfecting HEK293T cells, simultaneously adding pMD2.G (#12259; Addgene) for the virus envelope, pCMV‐dR8.2 dvpr (#8455; Addgene) for Gag and Pol genes, and polyethilenamine (#7854/100; R&D Systems). Supernatant from the HEK293T cells collected 48 and 72 h after transfection, containing lentivirus, was used at dilution 1 : 2, to transduce T24 and CAL‐29 cells. After 3 days, GFP+ cells were subsequently sorted and expanded.

### 2.3 Reagents

TAK‐228 (S2811), TAK‐117 (S8581), everolimus (S1120) (Selleckchem, Houston, TX, USA) and tunicamycin from Streptomyces (T7765‐5 mg; Sigma‐Aldrich) were prepared as 10 mm solutions in DMSO. Recombinant human‐interferon‐gamma protein (IFNγ) (285‐IF‐100; Bio‐Techne R&D Systems, Minneapolis, MN, USA) was dissolved at 0.2 mg·mL^−1^ in distilled water, and IFNβ‐1a (Merck, Rahway, NJ, USA; Rebif 44 μg/0.5 mL, L67R0901A). Recombinant human EGF (324831; Calbiochem, Merck Millipore, Darmstadt, Germany) was dissolved at 100 μg·mL^−1^ in PBS 0.1% BSA. Stock solutions were aliquoted and stored at −20 °C. Cetuximab (Erbitux; Merck; 5 mg·mL^−1^) and atezolizumab (Roche, Basel, Switzerland; 60 mg·mL^−1^) were provided by the Hospital del Mar pharmacy (Barcelona, Spain) and was prepared in physiologic serum and was stored at 4 °C. Anti‐αIFNAR2: Anti‐α‐Interferon Receptor Chain 2 Mouse mAB (MMHAR‐2) (407295; Merck‐Life Science, Darmstadt, Germany) was dissolved at 500 μg·mL^−1^ in PBS 0.1% BSA and was stored at −80 °C. Drugs and cytokines were used at doses indicated in the figure legends.

### 2.4 Quantitative real‐time polymerase chain reaction (qRT‐PCR)

Total RNA was extracted from cells, in basal conditions or after treatment using RNeasy kit (74104; Qiagen, Venlo, The Netherlands). cDNA was synthetized using random primers and High‐Capacity cDNA Reverse Transcription Kit (Applied Biosystems, Foster City, CA, USA). cDNA's samples were amplified using specific primers and LightCycler® 480 SYBR Green I Master (4707516001; Roche Diagnostics) in the QuantStudio™ 12K Flex (Applied Biosystems™). Samples were loaded in triplicate and primer amplification was done at 57 °C or 60 °C. Human primer pair sequences (*CD274* [12], *IFNB1* [13], *EGF* [14], *HIF1A*, *IL6*, *GAPDH*, *ATP5F1E*) are shown in Table S1. Gene expression was calculated as 2 to the power of −ΔΔCt, where ΔΔCt = (Ct_Gene_ − Ct_ATP5E or GAPDH_) Assay.

### 2.5 Western blotting

Western blots were performed according to standard protocols. The following antibodies were used: PD‐L1 (13684S), EGFR (#2232), pS6 (Ser235/236) (#2211), S6 (#2212), pGSK3α/β (Ser21/9) (#9331) and GADPH (#5174) from cell signaling, HLA class I ABC (ab70328) and IFNGR1 (ab134070) from Abcam, Cambridge, UK and GSK‐3β (sc‐9166) from Santa Cruz Biotechnology, Inc., Dallas, TX, USA. Mouse and rabbit horseradish peroxidase (HRP)‐conjugated secondary antibodies (GE Healthcare Life Sciences, Marlborough, MA, USA). GAPDH antibody was used as control to verify equal protein loading across samples. Bands were measured using imagej software, Bethesda, MD, USA. In all the figures, representative blots from independent experiments are shown.

### 2.6 Detection of the PD‐L1 and HLA‐I on cell surface by flow cytometry

Cells were harvested and then, incubated with the indicated antibodies: PD‐L1 (anti‐human CD274‐PE [B7‐H1], 12‐5983‐42; Thermo Fisher, eBioscience, San Diego, CA, USA) or isotype (mouse IgG1 kappa Isotype control [clone P3.6.2.8.11], 12‐4714‐42; Thermo Fisher, eBioscience), HLA‐I (anti‐human HLA‐ABC, clone G46‐2.6‐Mouse IgG1, k‐FCM‐APC, 555555; BD‐Biosciences, San Jose, CA, USA) or isotype (mouse IgG1, APC, MA5‐18093; Fisher Scientific) diluted in PBS 2% FBS at 4 °C for 30 min. Then, cells were washed three times with PBS 2% FBS. DAPI (D1309; ThermoFisher Scientific, Waltham, MA, USA) was added at a concentration of 1 μg·mL^−1^. Stained cells were analyzed using BD LSR Fortessa 4L 360 Cell Analyzer (647788) for PD‐L1 and LSRII 4L 360 Cell Analyzer for HLA‐I. Results were analyzed and plotted with flowjo software, Ashland, OR, USA. The MFI ratio (mean fluorescence intensity ratio) for each condition was obtained by performing the following calculation: mean fluorescence intensity for PD‐L1 or HLA‐I/mean fluorescence intensity isotype.

### 2.7 *CD274* (*PDL1*) copy number alterations (CNAs) determined by fluorescence *in‐situ* hybridization (FISH)

FISH analysis was carried out based on our previous experience [15]. FISH was performed in fixed material obtained from the cells after application of a conventional cytogenetic protocol. For analysis of CNAs ZytoLight® SPEC CD274, PDCD1LG2/CEP9 Dual Color Probe (ZytoVision, Bremerhaven, Germany) were employed. Gene amplification was defined as mean gene/mean centromere ratio ≥ 2.0.

### 2.8 Viability assays

Three different types of assays were used, depending on the needs of each experiment. In most experiments, the crystal violet assay was used. Briefly, bladder cancer cells were seeded at a density depending on their doubling time in 24‐well flat bottom plates. After 24 h, cells were treated as indicated in figure legends. At the end of the experiments, cells were washed with PBS and cell viability was determined by crystal violet staining assay or luciferase assay. Cells were stained with crystal violet solution (10% acetic acid, 10% absolute ethanol, and 0.06% crystal violet) for 1 h. After that, the plates were washed with PBS and allowed to dry. Images of each plate were scanned and quantified using fiji imagej software, Bethesa, MD, USA (RRID: SCR_002285) and plotted as arbitrary units. For experiments involving bladder cancer cells expressing luciferase, viability was measured using the Luciferase assay System from Promega, Madison, WI, USA (E1500), which rapidly detects firefly luciferase activity following the manufacturer's protocol.

Cell viability of PBMC after the treatment with TAK‐228 was measured by the MTS CellTiter 96 AQ One solution Cell proliferation assay from Promega (G3581) following the manufacturer's protocol.

### 2.9 PD‐L1 expression analysis by IHC on patient‐derived explants (PDE) treated *ex vivo*

To analyze PD‐L1 expression in human tumor samples patient‐derived explants (PDE) [16] were used. Tumor tissue from patients with BC was processed as described in [17] following CEIm‐Parc de Salut Mar approval (2016/6767/I) and in accordance with the Helsinki Declaration. Tumor samples were obtained at Hospital del Mar (Barcelona) from June 2018 to October 2018. Donors were requested to provide specific and written consent. Fresh tumor samples were immediately sliced and cultured *ex vivo* with or without TAK‐228. Samples were formalin‐fixed paraffin‐embedded (FFPE), stained for PD‐L1 IHC 22C3 pharmDx in an Autostainer Link 48 platform, and assessed by an expert pathologist. Only tumor cells with linear membrane staining for PD‐L1 22C3, partial or complete and at any intensity, were included in the scoring. Non‐viable tumor cells without visible chromatin detail, including tumor cells with squamous differentiation showing pycnotic nuclei, were discarded. Immune cells within the tumor area were excluded from the scoring. Samples with less than 100 viable tumor cells were also discarded. Staining cells in areas of necrosis or extravasation were not considered toward the scoring.

### 2.10 PD‐L1 expression analysis by IHC on xenograft‐derived explants treated *ex vivo*

PD‐L1 expression was analyzed in tumor fragments originated from tumors xenografts generated by injecting CAL‐29 or T24 cells into mice. Tumors were generated in 5‐week‐old male BALB/c nude mice (from Charles River Laboratories) by subcutaneously injecting CAL‐29 (20 × 10^6^) or T24 (20 × 10^6^) cells mixed 1 : 1 with Matrigel. After excision, the tumor fragments were treated *ex vivo* with TAK‐228 and embedded in FFPE material. PD‐L1 staining was analyzed following the same protocol as patient‐derived explants described above. Animals were housed in the pathogen‐free animal facility at the Barcelona Biomedical Park (PRBB). All animal work was conducted according to protocol (JAM‐17‐0044‐P1) approved by the Generalitat de Catalunya and following the PRBB Institutional Animal Care and Scientific Committee guidelines.

### 2.11 Co‐culture of peripheral blood mononuclear cells (PBMC) or CD8+ cells with cancer cell lines

Tumor cells were seeded at a density depending on their doubling time in 24‐well flat bottom plates. Cells were treated with TAK‐228 or atezolizumab. Doses and times are indicated in figure legends. Peripheral blood mononuclear cells (PBMC) (with activated CD8+ cells) were added to the tumor cells in different E : T (effector : target) ratios, as indicated in the figure legends. At the end of the experiment, PBMC were eliminated, and cancer cell viability was determined by crystal violet staining or the luciferase assay, as described in ‘viability assays’ in Section 2.

For these experiments, PBMC were isolated from buffy coats of healthy donors obtained between 2020 and 2024 from the Blood and Tissue Bank of Catalonia, a public agency under the Department of Health of the Government of Catalonia. Donors were requested to provide specific and written consent. Buffy coats were processed following CEIm‐Parc de Salut Mar approval (2021/9967/I) and in accordance with the Helsinki Declaration. PBMC were purified via centrifugation over ficoll. PBMC were cultured in RPMI supplemented with l‐glutamine (2 mm·L^−1^), penicillin/streptomycin (100 U/100 μg·mL^−1^), 10% FBS and IL‐2 0.3 ng·mL^−1^ (PHC0021; Fisher Scientific) and maintained at 37 °C under a 5% CO_2_ humidified atmosphere. PBMC were incubated 24 h with dynabeads human T‐activator CD3/CD28 (111.31D; Thermo Fisher) which activates human T lymphocytes. These activated cells were then added to the tumor cells. CD69 expression was analyzed by flow cytometry in the CD8+ T lymphocytes to prove its activation. In addition, PD‐1 expression was analyzed in the CD8+ T lymphocytes. The following antibodies were used: Live/Dead Fixable violet (405 nm) (L34955) from Thermo Fisher, CD3 APC‐Cy7 (300426) from BioLegend, San Diego, CA, USA, CD4 HV500 (560768), CD8 FITC (561947) and CD69 PE‐Cy5 (555532) from BD Biosciences and PD‐1 PE (130‐117‐533) from Miltenyi Biotec, Bergisch Gladbach, Germany. After the incubation with antibodies, the cells were fixed with the BD cytofix/cytoperm fixation and permeabilization solution (BD 554722) from BD Biosciences. Stained cells were analyzed using BD LSR Fortessa TM Cell Analyzer (647788). Results were analyzed and plotted with flowjo software.

In selected experiments, co‐cultures of CD8+ T cells with cancer cell lines were performed. CD8+ T cells were isolated from PBMC using CD8+ T Cell Isolation Kit, human (130‐096‐495; Miltenyi Biotec) as described by the manufacturer. Once the CD8+ T cells were isolated, the experiments were performed following the same protocol as described for the PBMC.

### 2.12 Statistical analysis

Statistical analysis was carried out with spss version 22.0 (SPSS, Inc., Chicago, IL, USA). Differences in means between conditions were assessed using Student *t*‐tests with a null hypothesis of no group differences. Contrasts with *P* < 0.05 were considered statistically significant. Significant differences between treatment conditions are depicted according to different *P* value thresholds: *P < 0.05; **P < 0.01; ***P < 0.001 and ****P < 0.0001.

## 3 Results

### 3.1 Different constitutive PD‐L1 and HLA‐I expression levels in bladder cancer cell lines

**Fig. 1:**
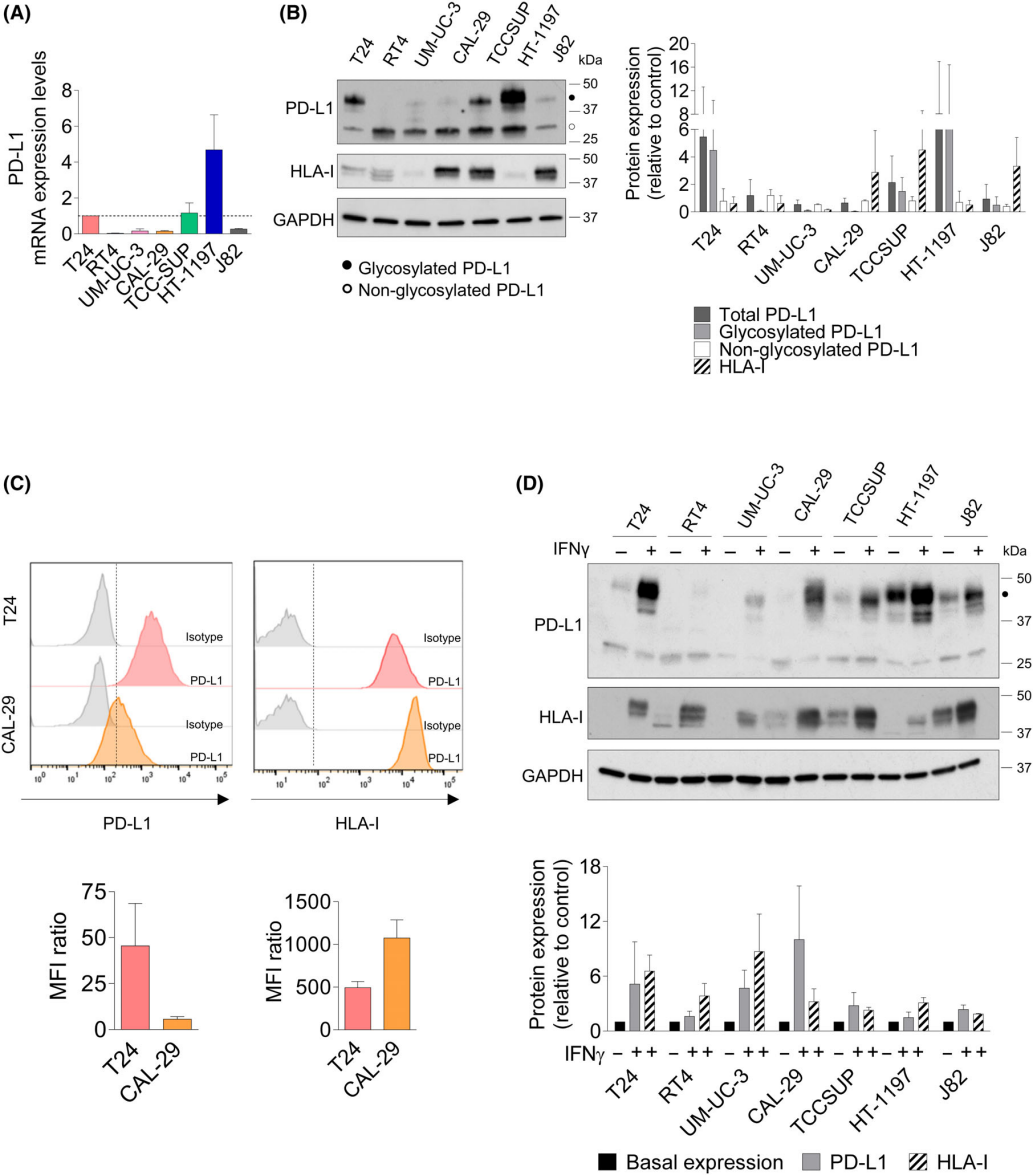
Characterization of the expression of PD‐L1 and HLA‐I in bladder cancer cells. (A–C) Cells were seeded in 100 mm^2^ dishes and left untouched for 48 h. (A) Basal mRNA expression of PD‐L1. PD‐L1 mRNA was detected by qRT‐PCR. mRNA levels were normalized to the T24 cells expression level (set at 1, dotted line). The average of two independent experiments is shown. (B) Basal expression of PD‐L1 and HLA‐I proteins. Cellular extracts were analyzed by western blot. The graph shows the basal levels of PD‐L1. The graph shows the levels of total PD‐L1, glycosylated PD‐L1, non‐glycosylated PD‐L1 and the basal levels of HLA‐I. The average of three independent experiments is shown. (C) Basal expression of PD‐L1 and HLA‐I in the cell surface of T24 and CAL‐29 cells. Graph shows the MFI ratio (mean fluorescence intensity ratio) for PD‐L1 (left) and HLA‐I (right) from each cell line. Representative image of the PD‐L1 (left) and HLA‐I (right) expression detected by flow cytometry from three independent experiments are shown. (D) Effect of IFNγ on PD‐L1 and HLA‐I protein expression. Cells were seeded and after 24 h the cells were treated with IFNγ (50 ng·mL^−1^) for 48 h. Cellular extracts were analyzed by western blot. The graph shows the levels of PD‐L1 and HLA‐I in each cell line, expressed as fold induction versus control arbitrarily set at 1. Every protein (PD‐L1 or HLA‐I) was quantified in relation to its baseline; however, we have only shown a basal condition in the graph to make the results easier to read. Representative blots from two independent experiments are shown. Error bands indicate standard deviation (SD).

We characterized the levels of PD‐L1 expression in 7 BC cell lines under basal conditions to identify intrinsic differences that could influence their immune evasion potential. First, we evaluated PD‐L1 mRNA expression by RT‐qPCR. T24 cells have previously been established as PD‐L1 expressors [18], and thus, we standardized our results against the mRNA expression of this cell line, which was arbitrarily set at 1. We considered expression to be high when values were ≥ 1. We found clear differences in basal PD‐L1 mRNA expression across the diverse cell lines. This distinction allowed us to categorize the cells into two distinct subsets: those with high PD‐L1 expression (T24, TCC‐SUP, and HT‐1197) and those with low PD‐L1 expression (RT4, UM‐UC‐3, CAL‐29, and J82). Notably, among these subsets, HT‐1197 cells exhibited the most prominent constitutive PD‐L1 over expression (Fig. 1A). Then, we examined the expression of PD‐L1 protein using western blotting and observed a correlation between PD‐L1 protein levels in whole‐cell lysates and mRNA levels (Fig. 1B). In our analysis, we identified two distinct bands, which potentially indicate the presence of both the glycosylated and non‐glycosylated forms of the PD‐L1 protein. As previously described, the upper band of ~ 50 kb corresponds to the glycosylated form present in the cell membrane, which is essential for PD‐L1 and PD‐1 interaction, while the lower band of ~ 30 kb corresponds to the non‐glycosylated form [19, 20]. To confirm this, we treated the T24 cells with an inhibitor of glycosylation (tunicamycin) and saw an increase in the non‐glycosylated form of the protein (Fig. S1A). Recognizing the importance of the glycosylated protein in the immune responses, we will now exclusively focus our efforts on studying the glycosylated form of PD‐L1 in western blots, as indicated by a black dot in the figures.

Combining the evaluation of PD‐L1 and major histocompatibility complex (MHC) class I, also known as HLA‐I, has been proposed as a potential biomarker for PD‐1/PD‐L1 inhibitor response in BC [21]. Downregulation of HLA‐I, is a key immune evasion mechanism linked to resistance to PD‐1/PD‐L1 inhibitors in cancer [22]. We analyzed HLA‐I levels in the same cell lines also in basal conditions. While all cell lines expressed HLA‐I, they could be categorized into two main groups: high HLA‐I‐expressors (CAL‐29, TCCSUP and J82 cells) and low HLA‐I‐expressors (T24, RT4, UM‐UC‐3 and HT‐1197 cells) (Fig. 1B).

We selected two cells with different PD‐L1 and HLA‐ I expression levels, T24 (high PD‐L1/low HLA I expression) and CAL‐29 (low PD‐L1/high HLA I expression), to confirm the presence of these proteins on the cell surface. Flow cytometry analysis of surface PD‐L1 and HLA confirmed the varied expression levels consistent with the observations from the western blot (Fig. 1C).

### 3.2 Genetic analysis of PD‐L1 gene (*CD274*) in bladder cancer cell lines

Genomic alterations affecting PD‐L1, such as copy number gains, may influence the expression levels of PD‐L1 across various cancer types [23]. The two cell subsets, high or low PD‐L1 protein expressors that we have identified, led us to assess the PD‐L1 gene status using FISH analysis, despite its limited clinical utility for diagnosing PD‐L1 in BC, as per previous knowledge [15]. Despite cells displaying trisomy or polysomy of chromosome 9, no gene amplifications or deletions were detected. Only the T24 cells exhibited a copy number gain of *CD274* (Fig. S1B). This suggests that the observed differences in PD‐L1 expression likely are not due to genetic alterations. Representative FISH analysis images of high PD‐L1 expressor (HT‐1197) and low PD‐L1 expressor cells (RT4) cells are shown in Fig. S1C. Subsequently, an exploration of the PD‐L1 gene status in tumor samples was undertaken. This assessment was performed *in silico* using cBioPortal [24], with data obtained from the TCGA PanCan 2018 and the MSK Our Urol 2017 public datasets. The results revealed that less than 5% of patients with BC exhibit PD‐L1 gene amplifications or mutations (Fig. S1D). Interestingly, a study found that *CD274* amplifications may serve as potential biomarkers for ICI response in UC [25].

### 3.3 IFNγ increases the levels of PD‐L1 in bladder cancer cell lines

We wanted to analyze whether IFNγ, a known transcriptional inducer of PD‐L1, affected PD‐L1 expression in different BC cell lines. When exposed to recombinant IFNγ, all cells exhibited increased glycosylated PD‐L1 expression. Even cells that did not display glycosylated PD‐L1 under basal conditions exhibited a slight elevation in glycosylated PD‐L1 expression upon exposure to IFNγ. The sole exception was RT4 cells, which showed no apparent protein increase (Fig. 1D). We then compared the effects of IFNγ on PD‐L1 mRNA expression in RT4 and CAL‐29 cell lines. IFNγ was effective in enhancing PD‐L1 mRNA expression in both cell lines (Fig. S1E). This suggests that the differences observed in RT4 might arise from post‐translational regulatory mechanisms impeding PD‐L1 expression. IFNγ also enhances HLA‐I expression to facilitate neoantigen presentation in tumor cells [26]. We confirmed that IFNγ increased HLA‐I in all tested cell lines (Fig. 1D).

### 3.4 TAK‐228 upregulates the levels of PD‐L1 in bladder cancer cells lines

We aimed to assess the effects of the inhibition of PI3K/AKT/mTOR pathway on PD‐L1 expression. Our investigation involved testing the effects of specific inhibitors: TAK‐228 (targeting mTORC1/2), everolimus (targeting mTORC1), and TAK‐117 (targeting PI3Kα). For this analysis, we selected T24 and CAL‐29 cell lines, characterized by high and low basal PD‐L1 expression levels, respectively. Cells were treated with these drugs at doses that effectively inhibit their respective targets (p‐S6, p‐4E‐BP1, p‐AKT for TAK‐228, p‐S6 for everolimus and p‐AKT for TAK‐117). This was demonstrated in a prior study by our group, where CAL‐29 cells showed high sensitivity to all three drugs while T24 responded only to mTOR inhibitors [17].

**Fig. 2:**
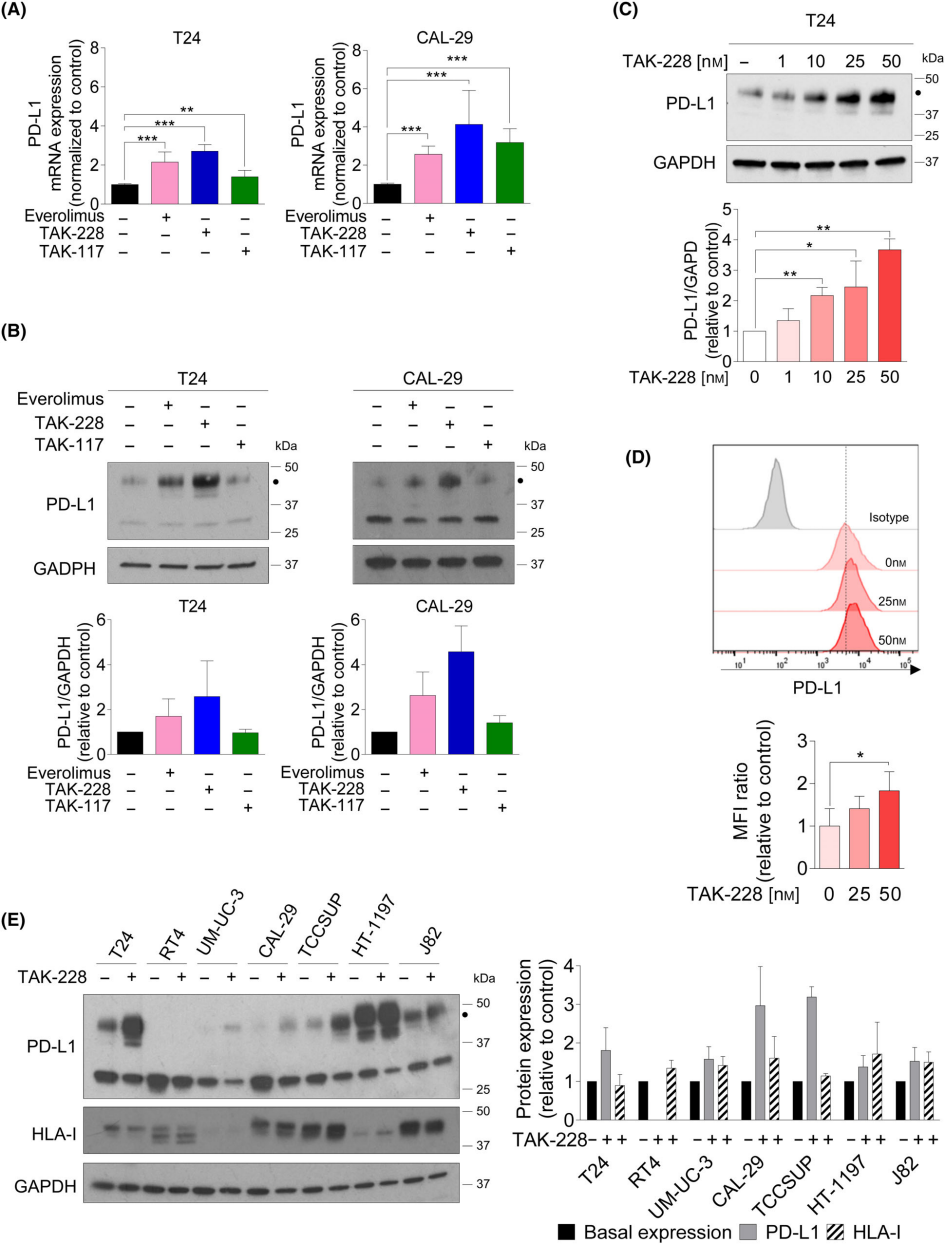
Characterization of the effects of PI3K/AKT/mTOR pathway inhibition on PD‐L1 regulation in bladder cancer cells. Cells were seeded in 100 mm^2^ dishes and 24 h later, cells were treated with everolimus (100 nm), TAK‐228 (50 nm) or TAK‐117 (3.5 μm) for 48 h. (A) Effect of the drugs on mRNA expression of PD‐L1. PD‐L1 mRNA was detected by qRT‐PCR. mRNA levels were normalized to the control cells expression level (set at 1). The average of three independent experiments is shown. (B, C) Effect of the drugs on PD‐L1 protein expression. Cellular extracts were analyzed by western blot. The graph shows the levels of PD‐L1 after the treatment, expressed as fold induction versus control arbitrarily set at 1. The average of three independent experiments is shown. The black dot indicates the glycosylated form of PD‐L1. (B) Effect of everolimus, TAK‐228 or TAK‐117 in T24 and CAL‐29 cells. (C) Effect of different doses of TAK‐228 (1, 10, 25, and 50 nm) on PD‐L1 expression in T24 cells. (D) Effects of TAK‐228 in the expression of PD‐L1 in the cell surface of T24 cells. The graph shows the fold‐change MFI ratio (mean fluorescence intensity ratio) for PD‐L1 relative to control cells. Representative image of the PD‐L1 expression detected by flow cytometry from three independent experiments are shown. (E) Effect of the TAK‐228 on PD‐L1 and HLA‐I protein expression in seven bladder cancer cells. The graph shows the levels of PD‐L1 and HLA‐I after the treatment in each cell line, expressed as fold induction versus basal conditions arbitrarily set at 1. The black dot indicates the glycosylated form of PD‐L1. Every protein (PD‐L1 or HLA‐I) was quantified in relation to its baseline; however, we have only shown a basal condition in the graph to make the results easier to read. Representative blots from two independent experiments are shown. Error bands indicate standard deviation (SD). Asterisks indicate significant differences between groups by Student *t*‐test (*P < 0.05, **P < 0.01 and ***P < 0.001).

Notably, both TAK‐228 and everolimus led to elevated PD‐L1 mRNA levels, indicating the potential impact of mTOR inhibitors on PD‐L1 transcriptional regulation (Fig. 2A). This increase in mRNA levels corresponded to a similar trend of increased glycosylated PD‐L1 form after treatment with these drugs (Fig. 2B). Conversely, while TAK‐117 increased PD‐L1 mRNA levels but had minimal effect on PD‐L1 protein levels under these conditions (Fig. 2A,B). Several factors could contribute to this phenomenon. These factors include the possibility that the induced increase in PD‐L1 mRNA levels might be counteracted by accelerated mRNA decay, which is influenced by microRNAs and RNA‐binding proteins. Alternatively, TAK‐117 may activate signaling pathways that indirectly affect PD‐L1 protein stability or degradation, possibly leading to opposing effects on mRNA and protein levels. However, this study did not investigate these specific molecular mechanisms, as its primary focus shifted to a deeper exploration of the impact of TAK‐228 on PD‐L1.

TAK‐228 increased the levels of PD‐L1 in a concentration‐dependent manner, with a noticeable rise starting at 10 nm in both CAL‐29 and T24 cell lines (Fig. 2C and Fig. S2A). Our primary focus is on glycosylated PD‐L1 levels, but it is important to note that the visibility of both glycosylated and non‐glycosylated PD‐L1 bands can vary among replicates and under different experimental conditions. Throughout our work, we have observed that consistency in the presence of both PD‐L1 forms is not always guaranteed. This variation can be attributed to factors such as the sensitivity of western blotting, which can affect the visualization of the less abundant non‐glycosylated form.

Subsequently, we confirmed through flow cytometry analysis that TAK‐228 indeed augmented the level of PD‐L1 on the cell surface of both cell lines (Fig. 2D and Fig. S2B). Our assessment extended to additional BC cell lines, wherein TAK‐228 consistently raised the levels of glycosylated PD‐L1. This effect was observed across most tested cells, irrespective of their baseline expression levels. Notably, the most significant increases were observed in T24, CAL‐29, and TCCSUP cell lines (Fig. 2E). Remarkably, similar to our observations with IFNγ treatment in Fig. 1, the PD‐L1 levels in RT4 cells remained largely unchanged. Additionally, we evaluated HLA‐I expression after TAK‐228 treatment. Across five models, a moderate increase in HLA expression was observed, significantly lower compared to the impact seen with IFNγ. HLA‐I expression remained almost unaffected in TCC‐SUP cells, while in T24 cells, there was a tendency for HLA‐I expression to decrease (Fig. 2E).

TAK‐228 or other mTORC1/2 inhibitors have been assessed in combination with the chemotherapeutic agent paclitaxel in over 10 clinical trials across various solid tumors. Among them, we led a phase II trial in which TAK‐228 was investigated for advanced UC in combination with paclitaxel (NCT03745911). Consequently, we analyzed PD‐L1 expression in BC cell lines treated with TAK‐228 and paclitaxel, recognizing the significant therapeutic potential of this combination. Our observations revealed that PD‐L1 levels were increased in this combination setting, although the increase was slightly less pronounced compared to the effect observed with TAK‐228 alone (Fig. S2C) indicating a detrimental effect on PD‐L1 with the concomitant use of chemotherapy.

### 3.5 TAK‐228 upregulates the levels of PD‐L1 in xenografts‐derived explants treated *ex vivo*

In the investigation of the impact of TAK‐228 on PD‐L1 in tumor samples, we utilized cell line (CAL‐29 and T24) derived xenograft samples from our previous work [17]. Similar to the findings with the cell lines, T24 tumor fragments exhibited higher PD‐L1 levels than CAL‐29 fragments under normal conditions. However, whenTAK‐228 was introduced, it increased PD‐L1 levels in CAL‐29 tumor cells, but decreased PD‐L1 staining in T24 fragments (Fig. S2D). It's important to note that the previous preclinical model exposed cells to TAK‐228 for 24 h [17], while this study extended the assessment to 48 h. Representative PD‐L1 staining images for CAL‐29 tumors, along with PD‐L1 staining percentages in tumor cells, are provided in Fig. S2D,E.

### 3.6 TAK‐228 increase the levels of PD‐L1 through different mechanisms

**Fig. 3:**
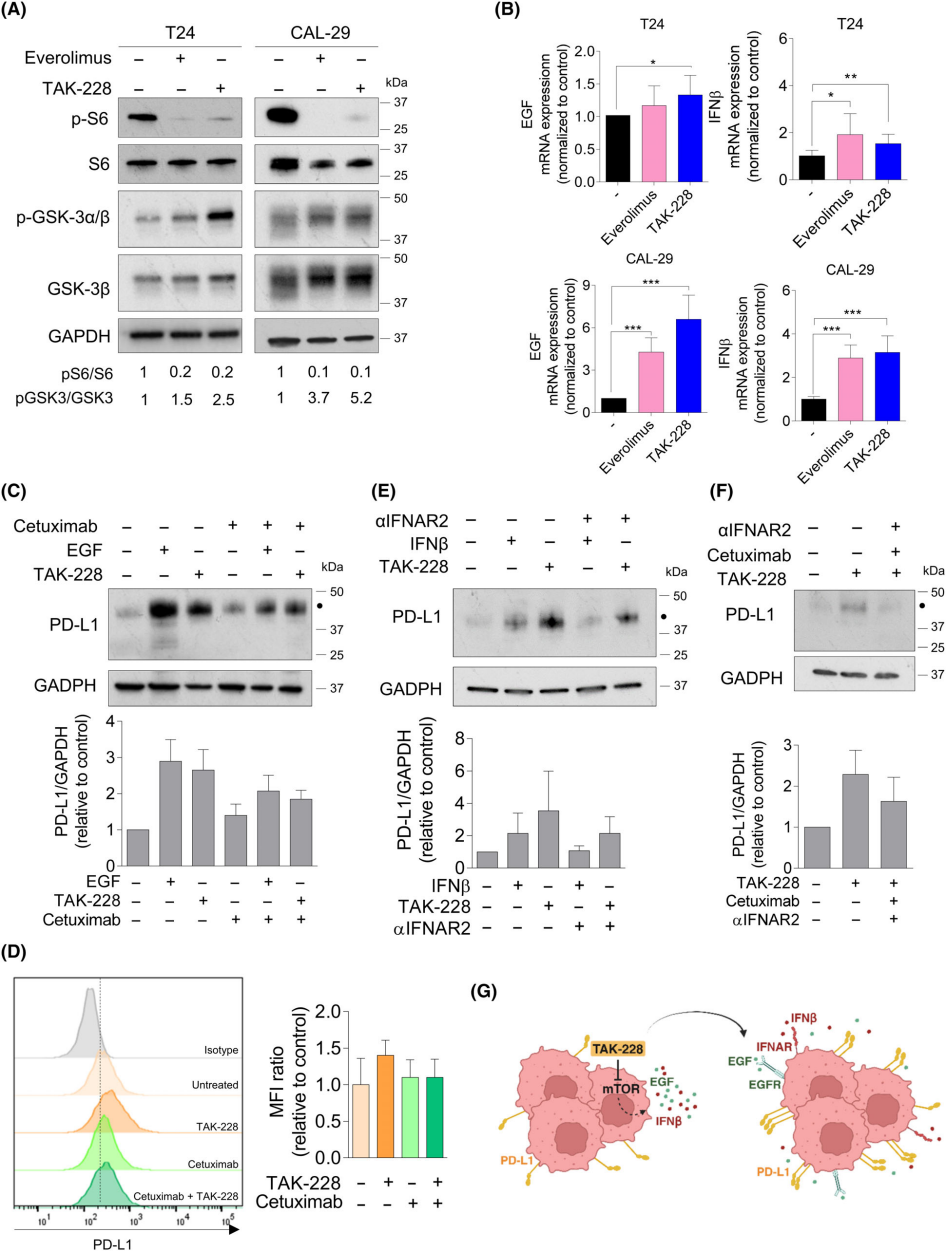
Possible mechanisms by which mTOR inhibitors might regulate PD‐L1. (A, B) Cells were seeded in 100 mm^2^ and 24 h later, cells were treated with everolimus (100 nm) or TAK‐228 (50 nm) for 48 h. (A) Effect of the drugs on S6 and GSK3 protein expression. Cellular extracts were analyzed by western blot using the indicated antibodies. The quantifications show the ratio between phosphorylated and total protein in each condition, expressed as fold induction versus control arbitrarily set at 1 from three independent experiments. (B) Effects of the drugs on the mRNA expression of EGF or IFNβ in T24 (top) and CAL‐29 (below). The mRNA levels of EGF or IFNβ were analyzed by qRT‐PCR after the treatment with the drugs. mRNA levels were normalized to the control cells expression level set at 1. The average of three independent experiments is shown. (C, D) Effects of the blockage of EGFR by cetuximab in CAL‐29 cells. Cells were treated with cetuximab (15 μg·mL^−1^) for 3 h and then TAK‐228 (50 nm) or EGF (25 ng·mL^−1^) were added for 48 h. (C) Cellular extracts were analyzed by western blot. The graph shows the levels of PD‐L1 expressed as fold induction versus control arbitrarily set at 1 from three independent experiments. The black dot indicates the glycosylated form of PD‐L1. (D) The graph shows the fold‐change MFI ratio (mean fluorescence intensity ratio) for PD‐L1 relative to control cells. Representative image of the PD‐L1 expression detected by flow cytometry from three independent experiments. (E) Effects of the blockage of IFNAR by anti‐IFNAR2 in CAL‐29 cells. Cells were treated with anti‐IFNAR2 (0.4 μg·mL^−1^) for 3 h and then TAK‐228 (50 nm) and/or IFNβ (2700 U) was added for 48 h. Cellular extracts were analyzed by western blot. Values show the levels of PD‐L1 expressed as fold induction versus control arbitrarily set at 1 from two independent experiments. The black dot indicates the band of the glycosylated form of PD‐L1. (F) Effects of the dual blockage of EGFR and IFNAR in CAL‐29 cells. Cells were treated with anti‐IFNAR2 (0.4 μg·mL^−1^) and cetuximab (15 μg·mL^−1^) for 3 h and then TAK‐228 (50 nm) was added for 48 h. Cellular extracts were analyzed by western blot. Values show the levels of PD‐L1 expressed as fold induction versus control arbitrarily set at 1 from three independent experiments. The black dot indicates the band of the glycosylated form of PD‐L1. (G) Schematic representation of the proposed mechanism of action for TAK‐228. Illustration was created with BioRender.com. Error bands indicate standard deviation (SD). Asterisks indicate significant differences between groups by Student *t*‐test (*P < 0.05, **P < 0.01 and ***P < 0.001).

We next studied the mechanism behind the upregulation of PD‐L1 induced by the mTOR inhibitor TAK‐228 in the BC cell lines, comparing it with another mTOR inhibitor, everolimus. Previous research has indicated mechanisms that can enhance PD‐L1 levels in a post‐transcriptional manner. We examined two of these mechanisms. First, mTOR inhibitors are known to elevate PD‐L1 levels by inhibiting S6 phosphorylation, thereby enhancing PD‐L1 stabilization [27]. Our findings are aligned with this notion; TAK‐228 treatment led to decreased levels of phosphorylated S6 (Fig. 3A). The second described mechanism is based on the concept that inactivating GSK3β has been shown to stabilize and increase PD‐L1 in breast cancer cells [28, 29]. Our study revealed that TAK‐228 inactivated GSK3β by increasing its phosphorylation in Ser21/9, suggesting its involvement in PD‐L1 upregulation (Fig. 3A). Everolimus treatment yielded similar results.

We also examined key PD‐L1 expression regulators intrinsic to the tumor cells that might be influenced by the drug [30], including soluble factors released by tumor cells such as EGF (epidermal growth factor) [31], cytokines like IFNβ (interferon‐β) [32] and IL‐6 (interleukin‐6) [33] and transcription factors like HIF‐1α (HIF1A) (hypoxia‐inducible factor 1‐α) [34, 35]. It's worth noting that we did not examine whether TAK‐228 modifies the expression of IFNγ, a potent PD‐L1 inducer, as this cytokine is exclusively secreted by immune cells. qRT‐PCR was used to analyze the impact of TAK‐228 and everolimus on these factors. Both inhibitors significantly raised EGF and IFNβ mRNA levels in T24 and CAL‐29 cells (Fig. 3B). We also observed a significant increase on HIF1A and IL‐6 levels in CAL‐29 cells (Fig. S3A). IFNβ acts as a transcriptional regulator of PD‐L1 [30] while EGF induces PD‐L1 glycosylation [29, 30]. To better understand how these factors could influence PD‐L1 transcription in BC cells, we treated cells with EGF, IFNβ, TAK‐228 and IFNγ (as a positive control for PD‐L1 mRNA and protein upregulation). All treatments significantly elevated PD‐L1 mRNA levels (Fig. S3B). However, while IFNβ and TAK‐228 prominently increased mRNA levels, this effect was relatively minor with EGF treatment (Fig. S3B). Levels of PD‐L1 protein were comparably raised following treatment with EGF, IFNβ and TAK‐228 (Fig. S3B). These findings suggest that TAK‐228 might induce PD‐L1 in a multifactorial manner, potentially influenced by the cellular context. Our subsequent focus was on TAK‐228 and the shared factors, IFNβ and/or EGF, between CAL‐29 and T24 cells that might contribute to the PD‐L1 increase.

### 3.7 Upregulation of PD‐L1 by TAK‐228 is EGF and IFNβ‐dependent

We further examined EGF as a potential inducer of PD‐L1 expression. First, we evaluated the basal levels of the EGF receptor (EGFR) in the seven cell lines, detecting EGFR expression in all cells (Fig. S3C). To demonstrate our hypothesis, we focused on CAL‐29 cells with high EGFR levels. Blocking EGFR using the inhibitor cetuximab partially diminished the PD‐L1 increase induced by EGF. Interestingly, EGFR blockade also partially reverted the PD‐L1 upregulation induced by TAK‐228 (Fig. 3C).

Similar results were obtained when we performed the same experiment, evaluating surface PD‐L1 expression by flow cytometry (Fig. 3D and Fig. S3D). These results suggest that PD‐L1 upregulation induced by TAK‐228 might involve EGF, particularly in the CAL‐29 context. We then tested whether IFNβ could also be involved in PD‐L1 upregulation. To do that, we used αIFNAR2, an antibody that neutralizes the action of the human α‐interferon by binding to the chain 2 of the receptor and blocking the biological actions of type‐I interferons (IFNα and IFNβ). Interestingly, IFNAR blockade partially reverted the PD‐L1 upregulation induced by TAK‐228 (Fig. 3E). Finally, our data illustrated that dual blockade of EGFR and IFNAR2 prevented TAK‐228‐induced PD‐L1 upregulation. In this triple treatment, PD‐L1 levels closely resembled the control levels (Fig. 3F). These results suggest that TAK‐228 could upregulate PD‐L1 through distinct mechanisms, including EGF and IFNβ (Fig. 3G).

### 3.8 IFNγ enhances the anti‐proliferative effects and the expression of PD‐L1 and HLA‐I induced by TAK‐228

**Fig. 4:**
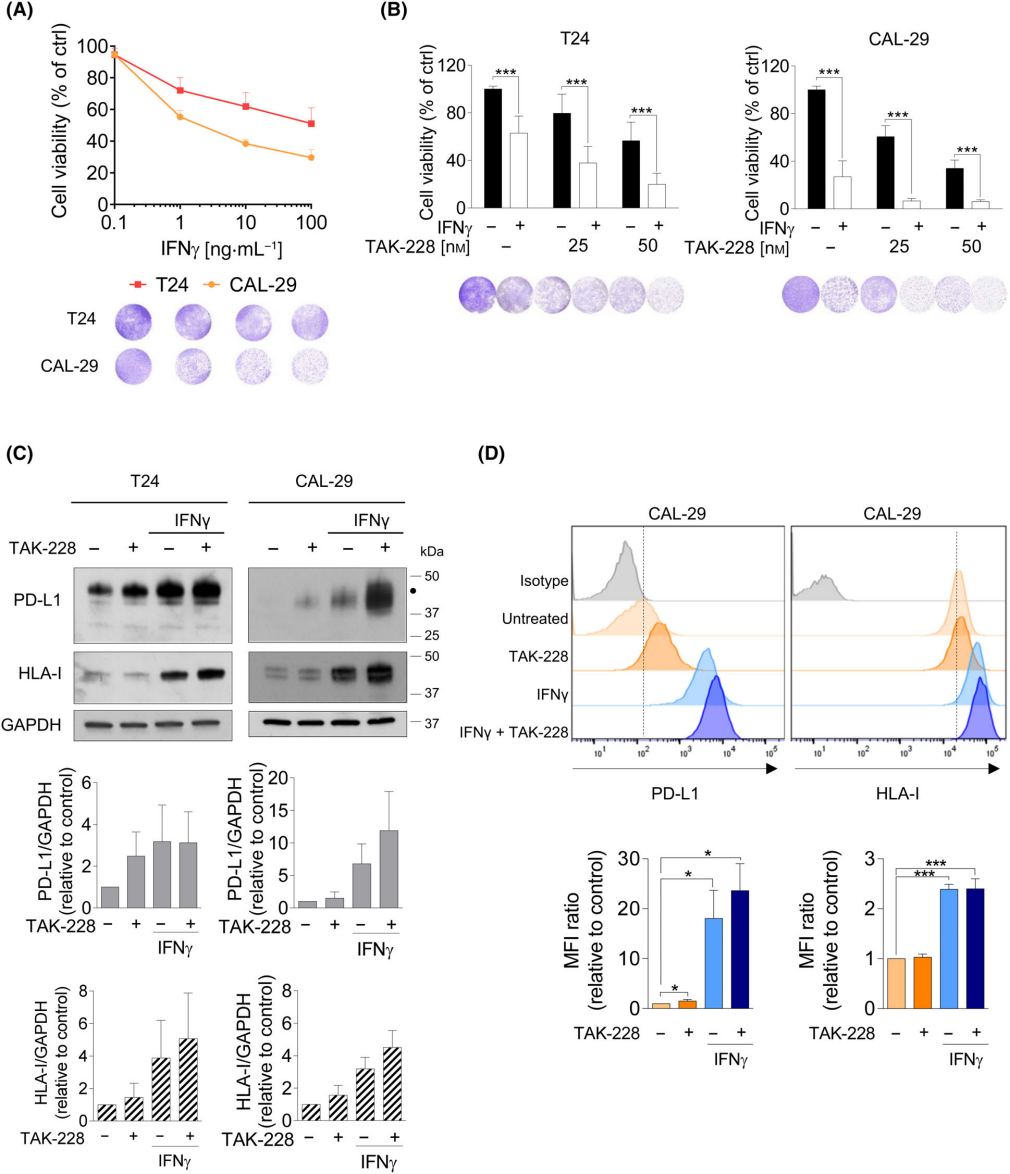
Characterization the effects of TAK‐228 in the presence of IFNγ on cell proliferation and on the regulation of PD‐L1 and HLA‐I in T24 and CAL‐29 cells. (A) Effects of the IFNγ on cell viability. Cells were seeded in 24‐well plate and 24 h later cells were treated with increasing doses of IFNγ (0.1, 1, 10 and 100 ng·mL^−1^) for 6 days. At the end of the experiment cells were washed with PBS and stained with crystal violet. Graphs show the percentage of cell viability relative to control from three independent experiments. (B) Effect of IFNγ in combination with TAK‐228 on cell viability. Cells were seeded in 24‐well plates and 24 h later cells were treated with TAK‐228 (25 and 50 nm), IFNγ (50 ng·mL^−1^) or the combination of both drugs for 6 days. At the end of the experiment cells were washed with PBS and stained with crystal violet. Graphs show the percentage of cell viability relative to control from three independent experiments. (C, D) Effect of IFNγ in combination with TAK‐228 on PD‐L1 and HLA‐I expression. Cells were seeded in 100 mm^2^ dishes and 24 h later cells were treated with TAK‐228 (50 nm), IFNγ (50 ng·mL^−1^) or the combination of both drugs for 48 h. (C) Total expression of PD‐L1 or HLA‐I in CAL‐29 cells. Cellular extracts were analyzed by western blot using the indicated antibodies. The graph shows the levels of PD‐L1 and HLA‐I in each cell line, expressed as fold induction versus control arbitrarily set at 1 from three independent experiments. The black dot indicates the glycosylated form of PD‐L1. (D) Expression of PD‐L1 or HLA‐I on the cell surface of CAL‐29 cells. The graph shows the fold‐change MFI ratio (mean fluorescence intensity ratio) for PD‐L1 (left) or HLA‐I (right) relative to control cells. Representative image of the PD‐L1 (left) or HLA‐I (right) expression detected by flow cytometry from three independent experiments. Error bands indicate standard deviation (SD). Asterisks indicate significant differences between groups by Student *t*‐test (*P < 0.05 and ***P < 0.001).

The tumor microenvironment can influence therapy response, and interactions between tumor cells, fibroblasts, and immune cells could be pivotal [36]. Our results indicated that TAK‐228 affects PD‐L1 and in some cell lines HLA‐I, both key immune response mediators. With this in mind, we aimed to explore whether TAK‐228 could influence the effect of the pro‐inflammatory cytokine IFNγ on UC cells. Notably, IFNγ led to reduced cell viability at 6 days, although T24 exhibited lower sensitivity to the effect of IFNγ compared to CAL‐29 cells (Fig. 4A). These differences might be due to distinct IFNγ receptor (IFNGR1) expression levels, as evident from cBioPortal database data indicating IFNGR1 mRNA expression differences between these cell lines (Fig. S4A) [24, 37]. Confirmed by western blot, CAL‐29 cells exhibited higher IFNGR1 protein levels compared to T24 cells (Fig. S4B). Interestingly, the addition of TAK‐228 notably amplified the cytotoxic impact of IFNγ on cell viability in both cell types (Fig. 4B). Analyzing PD‐L1 and HLA‐I levels post‐TAK‐228 treatment with or without IFNγ, we observed potentiated effects of TAK‐228 combined with IFNγ in both T24 and CAL‐29 cells, leading to significantly elevated intracellular levels of these two proteins observed through the analysis of whole‐cell lysate using western blotting (Fig. 4C). Additionally, the combination of IFNγ and TAK‐228 substantially increased the levels of surface PD‐L1. However, at the same time point surface HLA‐I levels were similar with the combination (Fig. 4D). Intricate processes govern the synthesis, transport, and surface display of HLA molecules. In specific conditions or treatments, these molecules can accumulate intracellularly before appearing on the cell membrane [38].

### 3.9 TAK‐228 affects the immune response in bladder cancer cell lines

**Fig. 5:**
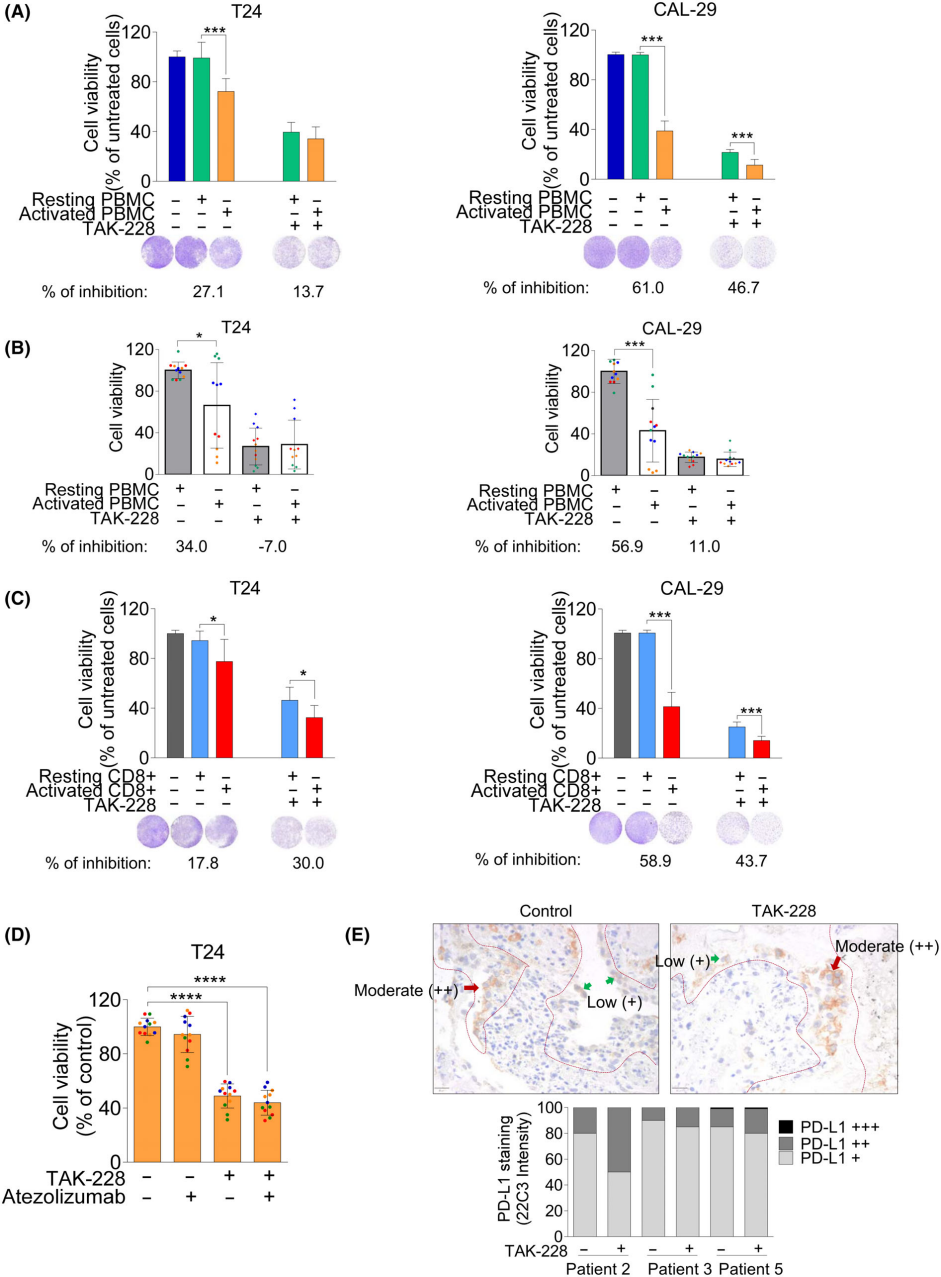
Effect of TAK‐228 on cell viability in a co‐culture system with PBMC (peripheral blood mononuclear cells) or CD8+ cells and tumor cells. (A–C) Cells were seeded in 24‐well plates and 24 h later cells were treated with TAK‐228 (50 nm). After 24 h, cells were co‐cultured with resting or activated PBMC in 10 : 1 (effector : target) ratio. After 72 h cells were washed with PBS and stained with crystal violet. Graphs show the percentage of cell viability relative to the cells treated with resting PBMC. % of inhibition in each condition = % of cell viability (resting PBMC) − % of cell viability (activated PBMC), considering 100% the resting PBMC condition in all the conditions. The experiments with CD8+ cells were performed as the PBMC experiments. (A) Effect of TAK‐228 on cell viability in the presence of PBMC. Blue column: control cells (without PBMC), green column: resting PBMC and orange column: activated PBMC. Graphs show the average of three different experiments. (B) Effect of TAK‐228 on cell viability in the presence of PBMC obtained from different healthy donors. Gray column: resting PBMC and white column: activated PBMC. PBMC from four healthy donors were used and the results of each one was represented in red, green, blue and orange dots. (C) Effect of TAK‐228 on cell viability in the presence of CD8+ T cells. Gray column: control cells (without CD8+), blue column: resting CD8+ and red column: activated CD8+. Graphs show the average of three different experiments. (D) Effect of TAK‐228 in combination with atezolizumab on cell viability in the presence of activated PBMC. T24‐GFP+‐Luc+ cells were seeded in 24‐well plates, and 24 h later, treated with TAK‐228 (50 nm). After 48 h, atezolizumab (10 μg·mL^−1^) was added. After 3 h, cells were co‐cultured with activated PBMC in a 10 : 1 ratio. After 72 h, the luciferase activity of T24 cells was analyzed with the luciferase assay system kit. Graphs show the percentage of cell viability relative to the control cells. Color dots represent the replicates of four different experiments. (E) Effect of TAK‐228 added *ex vivo* to patient‐derived explants. FFPE blocks were prepared from tumor fragments after 72 h of treatment with TAK‐228 [24.3 nm]. Tumor fragments were stained for PD‐L1. Representative IHC images of control and TAK‐228‐treated tumors (patient 2) were shown above. Necrotic areas, marked with the red dotted line, were excluded from the analysis and only PD‐L1 staining (in brown) in live tumor cells was quantified. Low intensity of PD‐L1: + (green arrow) and moderate intensity of PD‐L1: ++ (red arrow). Images were acquired at a magnification of 10 *×* 40× = 400×. Scale bar (20 μm) is shown on the bottom left of both images. Graph shows the distribution of the percentage of three patterns of PD‐L1 intensity: low (+), moderate (++) and high (+++) in basal conditions and after TAK‐228 treatment in samples from patients 2, 3 and 5. Patients 1 and 4 were not represented because no PD‐L1 staining was detected. Error bands indicate standard deviation (SD). Asterisks indicate significant differences between groups by Student *t*‐test (*P < 0.05, ***P < 0.001 and ****P < 0.0001).

To better explore the effects of TAK‐228 in the presence of the immune system, we co‐cultured tumor cells with PBMC from healthy donors previously activated with anti‐CD3/CD28. We confirmed PBMC's activation through flow cytometry analysis by measuring the percentage of CD69 expression in CD8+ cells (Fig. S5). We tested the effect of PBMC from three healthy donors on tumor cells using two E : T (effector : target) ratios (3 : 1 and 10 : 1) and two different time points (48 and 72 h). As a result of these experiments, we determined that the 10 : 1 ratio at 72 h was the most optimal for our research (Fig. S6A,B). Subsequently, we co‐cultured tumor cells with either resting or activated PBMC, and we added a control without PBMC. Resting PBMC had no notable impact on BC cell viability compared to control cells. Conversely, activated PBMC significantly reduced cell viability compared to resting PBMC‐treated cells (61.0% in CAL‐29 and 27.1% in T24 cells). This reduction in viability was attributed, at least in part, to activated CD8+ T cell cytotoxicity (Fig. 5A). Discrepancies in the response to activated PBMC may result from variations in either sensitivity to IFNγ secreted by T cells or antigen presentation. It is worth noting that T24 cells have lower HLA‐I levels compared to CAL‐29, even though this system lacks specific HLA‐I and TCR recognition.

Upon TAK‐228 treatment, the T cells cytotoxic impact was diminished. In CAL‐29, the percentage of cell inhibition decreased to 46.7% from 61.0% in the control. In T24, cell inhibition dropped to 13.7% from 27.1% in the control (Fig. 5A). Similar results were observed with a 5 : 1 (E : T) ratio (Fig. S6C). Taking all these data into account, TAK‐228 could affect the immune responses against tumoral cells. Given potential variations in immune responses among different individuals, we sought validation by replicating the experiments using PBMC from four distinct healthy donors. We confirmed differences in response between the different donors. Despite not finding significant differences, we observed a highly inhibition of the effect of PBMC in TAK‐228‐treated cells (Fig. 5B).

In parallel, we assessed an experimental condition where TAK‐228 and activated PBMCs were added simultaneously. Under this latter condition, we found no significant differences between the effects of TAK‐228 alone and its combination with PBMC treatment. These findings emphasize the importance of treatment timing (Fig. S6D). However, the impact of TAK‐228 on PBMC viability may have influenced these results, as shown in Fig. S6E.

Subsequently, our objective was to validate whether these effects were mainly due to the cytotoxic activity of CD8+ T cells. For that, we isolated the CD8+ cell population and we replicated the same experiment, yielding comparable outcomes (Fig. 5C).

### 3.10 The addition of an anti‐PD‐L1 antibody increased the efficacy of the immune system in eliminating TAK‐228 treated tumor cells

We studied if adding an ICI could change immune cells response toward TAK‐228‐treated tumor cells. We used luciferase‐expressing T24 or CAL‐29 cells, enabling precise detection of live tumor cells in co‐cultures with immune cells using a luciferase assay. Atezolizumab, an anti‐PD‐L1 inhibitor, was selected as the ICI. Initially, we tested its binding to PD‐L1 on tumor cell membranes using flow cytometry. Cells were stained with a fluorochrome‐labeled anti‐PD‐L1 antibody to analyze PD‐L1 expression. We observed that in the presence of atezolizumab, the labeled anti‐PD‐L1 antibody is unable to bind to PD‐L1, indicating atezolizumab binding to PD‐L1 (Fig. S6F).

We treated the cells with TAK‐228 for 48 h, followed by the addition of atezolizumab. Three hours later, activated PBMC were added for 72 h. A small decrease in cell viability was observed with atezolizumab in T24 cells (from 100% ± 6.3 in the control to 94.3% ± 13.3 with atezolizumab). However, atezolizumab alone did not affect CAL‐29 cells. The limited effects of atezolizumab may be explained by the low percentage of CD8+ cells expressing PD‐1+. We observed a variable range of CD8+ PD‐1+ cells within the different stocks used in these experiments, ranging from 5.13% to 39.3% (Fig. S5). However, we noted that atezolizumab enhanced the effect of CD8+ cells over TAK‐228 treated cancer cells (from 48.9% ± 8.8 with TAK‐228 to 43.9 ± 9.2 with the combination). Although the difference was only 5%, which was not statistically significant, we believe that the combination holds potential as a treatment for urothelial cancer (Fig. 5D). In the case of CAL‐29 cells, atezolizumab did not improve the effect of TAK‐228 (Fig. S6G).

### 3.11 TAK‐228 increased the expression of PD‐L1 in patient‐derived explants (PDE) treated *ex vivo*

We also studied the effect of TAK‐228 in patient‐derived explants consisting of human tumor tissue samples with different grading and staging from patients with BC. These samples were processed following procedures detailed in our prior work [17]. Each tumor sample (*n* = 5) was divided into two fragments: one treated with TAK‐228 and another left untreated as the control. We assessed PD‐L1 staining in these tumor samples (Table S2). These samples are characterized by small size, in order to not compromise diagnosis, and areas of necrosis. Nevertheless, all the cases included in the analysis had at least 100 viable tumor cells. We analyzed the presence of tumor cells expressing PD‐L1 in these specimens. Additionally, as TAK‐228 increases the level of PD‐L1 expression in cell lines, we wanted to explore whether this also occurred in tumor samples. We categorized PD‐L1 intensity into three patterns: low (+), moderate (++) and high (+++). Our evaluation focused solely on PD‐L1 staining in tumor cells. It is worth noting that in the clinical practice of analyzing PD‐L1 in UC patients, the intensity of staining is not defined, and immune cells are also examined. Nevertheless, our research focused on modifying PD‐L1 expression in cancer cells, and that was the type of analysis we required.

First, we analyzed basal PD‐L1 expression in the control samples, revealing two distinct groups of tumors: no PD‐L1‐expressors (patients 1 and 4) and PD‐L1‐expressors (patients 2, 3 and 5). In the non‐PD‐L1 expressor tumors, no positive PD‐L1 staining was observed in tumor cells after TAK‐228 treatment. Notably, assessment for Patient 1 was impracticable due to absence of enough viable tumor cells post‐TAK‐228 treatment. Of note, among these non‐PD‐L1 expressors, patient 1 had a G1 tumor (low‐grade tumor). In PD‐L1 expressing tumors, the predominant pattern of expression observed is low (+) PD‐L1 intensity staining in the majority of tumor cells (≈ 80–90%), with only a small fraction (≈ 10–20%) exhibiting moderate (++) PD‐L1 intensity staining. Following TAK‐228 treatment, we detected a decrease in the percentage of cells (≈ 50–80%) with low (+) staining and an increase in the percentage of cells (≈ 15–50%) with moderate (++) staining. These results indicated that TAK‐228 augmented the number of cells with moderately expressed PD‐L1 within this subgroup of tumors (Fig. 5E and Table S2). Importantly, these samples (*n* = 3) came from high‐grade tumors. Representative images are shown in Fig. 5E. Interestingly, these results mirror the *in vitro* results. Notably, RT4 cells, originating from a low‐grade tumor, exhibited minimal basal PD‐L1 expression that barely increased after TAK‐228 treatment. In contrast, cells from high‐grade tumors displayed PD‐L1 expression, which was further enhanced upon treatment with TAK‐228.

## 4 Discussion

PD‐1/PD‐L1 inhibitors are approved for advanced BC treatment [1]. These antibodies disrupt the PD‐1/PD‐L1 immune checkpoint pathway, enhancing T cell attack on tumors. Monotherapy responses vary, justifying the investigation of combination strategies to enhance efficacy [39]. The PI3K/AKT/mTOR is pivotal in certain tumors including UC, prompting interest in combining targeted therapies from this pathway with PD‐1/PD‐L1 inhibitors. In the current study, we have used BC models since it is one of the most prevalent types of UC cancer.

Preclinical research has shown efficacy of combining mTOR inhibitors with PD‐1/PD‐L1 inhibitors in syngeneic mouse models of renal cell carcinoma [40] oral cavity cancer [41], and colorectal cancer [42]. While this combination shows clinical interest in certain tumor types, its potential in advanced BC requires more investigation. To explore this, we studied the mTORC1/2 inhibitor TAK‐228, that has been proven effective in clinical trials in solid tumors [43, 44]. TAK‐228 has demonstrated effectiveness in combination with a PD‐L1 inhibitor in a preclinical model of colorectal cancer [45]. We have previously conducted investigations with TAK‐228 both in the preclinical setting [17] and in clinical trials (NCT03745911) [46] for BC.

Our present study is focusing on assessing the potential influence of TAK‐228 on anti‐tumor immune responses. It focuses into how TAK‐228 alters the interplay between cancer cells and immune cells in BC. Specifically, it examines the involvement of PD‐L1 and HLA class I molecules in this interaction. Our research has employed preclinical BC models, comparing TAK‐228 to other mTOR inhibitors as well as PI3Kα inhibitors. Dissecting PD‐L1 regulation in BC cells is crucial for understanding their response to treatments targeting the PD‐L1/PD‐1 pathway. We began by examining PD‐L1 expression in BC cell lines. Elevated PD‐L1 on cancer cells strongly suppresses the immune system reaction, but this overexpression can be counteracted by the PD1/PD‐L1 inhibitors. Our research has focused on the tumor microenvironment conditions with the cytokine IFNγ, secreted by activated immune cells such as T cells and natural killer (NK) cells. IFNγ elicits both immune‐activating and immunosuppressive effects [47]. It is well known that IFNγ binding to its receptors on cancer cells can triggers the IFNγ/JAK/STAT pathway that can result in increased PD‐L1 expression as a defense mechanism against T cell attacks in most tumors [48]. We exposed the cells to recombinant IFNγ. Remarkably, all BC cells demonstrated elevated PD‐L1 protein levels upon exposure to IFNγ, with the exception of RT4 cells. The distinct behavior of these cells may arise from translational or post‐translational regulatory mechanisms that impede its expression as our findings showed increased PD‐L1 mRNA levels in RT4 cells upon IFNγ exposure.

Our study revealed that TAK‐228 enhanced PD‐L1 levels in all examined BC cell lines. The extent of this increase was dependent on factors like the cell lines' origin (from low‐ or high‐grade tumors), their stage, and genetic background. RT4 cells from low‐grade tumors did not show PD‐L1 increase with TAK‐228, while cells like T24 or CAL‐29 derived from high‐grade tumors displayed upregulation of PD‐L1 in response to TAK‐228 treatment.

We further validated our *in vitro* results using *ex vivo* experimental models, including patient samples. As seen in cell lines, our investigation of tumor samples revealed two distinct groups of patients characterized by PD‐L1 expression. While drawing conclusions with the limited number of samples analyzed is questionable, it is important to note that TAK‐228 consistently increased PD‐L1 levels in the majority of the tumor samples analyzed in this study, suggesting potential translational relevance for clinical applications. Further studies with additional samples are needed to confirm these findings.

Although the increase in PD‐L1 expression caused by TAK‐228 might aid initially immune evasion, it might also be targeted therapeutically with PD‐1/PD‐L1 inhibitors. As a consequence, enhancing PD‐L1 expression through the use of mTOR‐targeted therapy might enhance the efficacy of ICI. PD‐L1 is vital for immune response suppression; its binding to PD‐1 on T cells hampers their cancer‐killing ability. Anti‐PD‐L1 antibodies reverse this, enabling robust T cell attacks. In fact, low PD‐L1 expression serves as a criterion for excluding the use of this therapy in UC patients.

Our approach of giving mTOR inhibitors to UC patients might enhance tumor recognition and destruction, potentially improving the overall efficacy of ICI. Patients with initially low PD‐L1 tumors could become responsive after mTOR inhibitor therapy‐induced PD‐L1 upregulation. In light of our experimental findings, this phenomenon can also be observed when BC cells are exposed to both TAK‐228 and exogenous IFNγ *in vitro*. TAK‐228 amplifies the effect of IFNγ on PD‐L1. While the effect of IFNγ alone may be sufficient it could be particularly valuable in poorly infiltrated tumors, where the spatial and temporal distribution of IFNγ secreted by activated CD8+ T cells within the tumor microenvironment is limited [49], resulting in areas with low IFNγ concentration. In such cases, TAK‐228 PD‐L1 enhancement could be physiological important.

Another potential beneficial action of TAK‐228 is related to the fact that active T cells and NK cells not only release cytolytic granules containing perforin and granzymes to eliminate cancer cells but also secrete IFNγ, which can trigger apoptosis in cancer cells [50]. Our results have shown that TAK‐228 also holds therapeutic potential by enhancing IFNγ's cytotoxic effects on cancer cells, thereby reinforcing the immune response against cancer. This underscores TAK‐228's potential to enhance immunotherapies targeting improved T cell cytotoxic activity against cancer cells.

Regarding the different mTOR inhibitors, we found that both TAK‐228 and everolimus can effectively increase PD‐L1 protein expression. However, the PI3Kα inhibitor (TAK‐117) raised the PD‐L1 mRNA levels but it did not lead to a subsequent protein increase. This discrepancy suggests unique roles for these inhibitors in the PD‐L1 pathway. Our work supports a previous study in which TAK‐228 and other mTOR inhibitors were found to increase PD‐L1 in lung cancer cell lines as well as other cancer cell types [27]. Also, another study reported increased PD‐L1 after the treatment with the mTORC1 inhibitor everolimus in renal cancer cells [40]. Conversely, other studies showed reduced PD‐L1 levels with PI3K, mTORC1 or mTORC1/2 inhibitors in lung cancer cells [51] that were associated with mTOR inactivation or AMP‐activated protein kinase (AMPK) activation, both sensors of the nutrient and energy status of the cell. Another study in NSCLC also demonstrated PD‐L1 inhibition with an mTORC1/2 inhibitor mediated in part by mTORC2/AKT/GSK3β‐dependent proteasomal degradation [52]. Additionally, another study showed a slightly decrease in PD‐L1 expression following treatment with the mTORC1 inhibitor rapamycin in triple‐negative breast cancer [53]. Importantly, it is worth noting that in these studies, the assessment of PD‐L1 expression occurred at a different time point than in our research. PD‐L1 expression was evaluated at 24 h or less. It's possible that at such an early time point, PD‐L1 synthesis or stabilization may not have fully occurred. These findings underscore the complexity of the relationship between mTOR signaling and PD‐L1 expression, which may be influenced by several status, the specific mTOR inhibitor utilized, and the context of the study.

We investigated deeper into the mechanisms behind the TAK‐228‐related enhanced PD‐L1 expression. The regulation of PD‐L1 is complicated, involving various aspects such as genetics, epigenetics, transcription, translation, and post‐translational factors [54, 55]. We focused our investigation on well‐established pathways associated with PD‐L1 stabilization, including the inhibition of S6 phosphorylation [27] and GSK3β inactivation [28]. Additionally, we explored alternative mechanisms and discovered that TAK‐228 enhances PD‐L1 expression through increased mRNA levels and the release of EGF and IFNβ. EGF, a vital growth factor, binds to EGFR on cancer cells, activating MAPK/ERK and PI3K/AKT pathways, ultimately promoting PD‐L1 gene transcription [31]. IFNβ, part of the interferon cytokine family, activates JAK–STAT signaling, upregulating PD‐L1 via interferon‐stimulated response elements (ISREs) in the gene promoter region [32]. Targeting these pathways could reduce PD‐L1 expression, making EGFR‐altered UC more susceptible to immune attacks [56]. Understanding interactions like EGF‐EGFR's influence on PD‐L1 is especially relevant given the ongoing clinical trials assessing the use of afatinib (EGFR‐TKI) in advanced BC (NCT02122172).

A recent study [45] revealed a cap‐independent translational mechanism for PD‐L1 upregulation via TAK‐228. Together with our discovery of TAK‐228‐induced EGF and IFNβ contributing to PD‐L1 upregulation in bladder cancer cells adds to understanding multiple mechanisms modulating PD‐L1 during mTOR inhibition by TAK‐228. Aligning with our findings, their study in colon cancer [45] suggests that increased PD‐L1 due to mTOR inhibition leads to immune suppression and resistance to mTOR inhibitors. In CT26 and MC38 syngeneic colon tumor models, TAK‐228 slowed tumor growth but increased PD‐L1 and reduced TILs. Combining anti‐PD‐L1 antibodies with TAK‐228 restored TILs, significantly enhancing TAK‐228 efficacy against tumor growth. These findings emphasize targeting PD‐L1 to improve mTOR‐targeted therapy. However, these immune effects might be context‐dependent and might vary based on dosage and treatment schedules.

Increasing PD‐L1 expression and enhancing antigen presentation via HLA‐I has been proposed as a potential strategy to make tumors more responsive to PD‐1/PD‐L1 inhibitors [36, 57]. T cell therapies often face challenges due to limited tumor‐specific antigen presentation, aggravated by HLA‐I downregulation as a mechanism of resistance. Our study demonstrated, that TAK‐228 increases intracellular HLA‐I molecules in tumor cells, especially in the presence of IFNγ. Similar results have been seen with various targeted therapies like RET and ALK inhibitors [58], EGFR inhibitors [59], CDK4/6 inhibitors [60] and MEK inhibitors [61] which are also able to increase HLA‐I. Some studies have shown the potential to enhance T cell‐based immunotherapies through HLA‐I upregulation, improving tumor antigen presentation and immune recognition [62, 63]. Our findings further support the promising role of combining TAK‐228 with ICI in patients with advanced BC.

We conducted co‐cultivation experiments to explore how the modulation of PD‐L1 and/or HLA‐I by TAK‐228 treatment impacts the response of BC cells to immune cell activity. These responses play a pivotal role in determining the overall effectiveness of anti‐tumor treatments [36]. In these experiments, we co‐cultured CAL‐29 or T24 cells with either PBMC or purified CD8+ T cells, both in their basal state and after pre‐treatment with TAK‐228. Under basal conditions, these cell lines exhibited distinctive responses. Notably, CAL‐29 cells displayed an increased sensitivity to immune cell‐induced cytotoxic effects and in experimental models, pre‐treatment with TAK‐228 demonstrated a significant ability to diminish the cytotoxic effects induced by CD8+ T cells. This observation strongly implies that the modulation of immune responses can be ascribed to the influence of TAK‐228 on the upregulation of PD‐L1. The addition of an anti‐PD‐L1 agent increased the cytotoxic effects of PBMC in the presence of TAK‐228 in T24 cells suggesting a potential avenue for combining dual mTOR inhibitors and anti‐PD‐L1 in clinical settings. However, recent reports from the phase II BISCAY trial (NCT02546661) have revealed no clinical benefits from the combination of durvalumab (anti‐PD‐L1) and vistusertib (mTOR1/2 inhibitor) for advanced UC [64], despite patient selection based on specific tumor genomic alterations expected to respond mTOR inhibitors. Nonetheless, although our analysis is not exhaustive, it underscores the potential for improvements in clinical trial design. Our findings suggest that TAK‐228 and anti‐PD‐L1 agents could be a promising combination using the appropriate treatment scheme, such as administering the mTOR inhibitor pre‐treatment before anti‐PD‐L1 treatment. Additionally, evaluating the presence of immune cell infiltration in tumors as potential targets for the anti‐PD‐L1 effect, could also substantially enhance the overall clinical effectiveness of this approach. Considering all our findings, the potential to increase tumor susceptibility to PD‐1/PD‐L1 inhibitors through PD‐L1 upregulation, enhanced antigen presentation via HLA‐I, and other influencing factors (amplifying IFNγ's cytotoxic effect) support the promising role of combining TAK‐228 with ICI in patients with advanced BC. Identifying biomarkers linked to PD‐L1 upregulation due to TAK‐228's mTOR inhibition, and connecting them with clinical responses, is essential to improve treatment outcomes. Our findings demonstrate that cancer cells exposed to TAK‐228 upregulate PD‐L1 expression, justifying exploring the synergistic addition of a PD‐L1/PD‐1 inhibitor in combination in the clinic.

## 5 Conclusions

Our preclinical findings in bladder cancer models highlight that TAK‐228 functions not only as an anti‐tumor agent but also triggers changes within tumor cells, such as *de novo* protein synthesis and adjustments in cell surface molecules like PD‐L1. These changes could potentially lead to immunosuppression and eventual resistance to mTOR inhibitors over time. These results hold clinical significance, encouraging deeper investigations into combining mTOR inhibitors with PD‐1/PD‐L1 inhibitors to enhance anti‐tumor immunity among BC patients.

## Conflict of interest

J. Bellmunt has served in consulting or advisory roles for Astellas Pharma, AstraZeneca/MedImmune, Bristol Myers Squibb, Genentech, Novartis, Pfizer, Pierre Fabre, and the healthcare business of Merck KGaA, Darmstadt, Germany; has received travel and accommodation expenses from Ipsen, Merck & Co., Kenilworth, NJ, and Pfizer; reports patents, royalties, other intellectual property from UpToDate; reports stock and other ownership interests in Rainier Therapeutics; has received honoraria from UpToDate; and has received institutional research funding from Millennium, Pfizer, Sanofi, and the healthcare business of Merck KGaA, Darmstadt, Germany. The rest of authors declare they do not have any conflict of interest.

## Author contributions

AH‐P, AR, and JB contributed to conception and design of the study. AH‐P, LC, MQ, OA‐L, SM, LS‐J, and FGQ contributed to development and setup of methodology for western blot, qRT‐PCR, viability assays, and co‐culture experiments. SM, FR, and NJR contributed to IHC and staining analysis. AR‐V, JA, and NJ‐R contributed to recruitment of patients and collection of clinical data. MS contributed to FISH analysis and interpretation. MG, AM, EA, and AH‐P contributed to flow cytometry panel design, flow cytometry analysis, and interpretation of immune experiments. AH‐P, LC, MQ, OA‐L, SM, LS‐J, FGQ, MG, NJ‐R, AR, and JB contributed to interpretation and discussion of the results. AH‐P, AR‐V, FR, JA, AR, AM, and JB contributed to writing, review, and/or revision of the manuscript. AR and JB contributed to study supervision. Each author provided feedback on the manuscript.

### Peer review

The peer review history for this article is available at https://www.webofscience.com/api/gateway/wos/peer‐review/10.1002/1878‐0261.13699.

## Supporting information


**Fig. S1.** Characterization of the expression of PD‐L1 in bladder cancer cells.
**Fig. S2.** Effects of TAK‐228 alone or in combination on PD‐L1 in cells and/or in *ex vivo* cells derived xenografts.
**Fig. S3.** Mechanism of PD‐L1 regulation by mTOR inhibitors.
**Fig. S4.** Expression of IFNGR1 in bladder cancer cells.
**Fig. S5.** Analysis of the activation of CD8+ T cells in PBMC.
**Fig. S6.** Co‐culture experiments with PBMC and tumor cells.
**Table S1.** Oligonucleotide sequence of primers used for qRT‐PCR.
**Table S2.** Effect of TAK‐228 in patient‐derived explants (PDE) treated *ex vivo*. Clinical information and results of the IHC staining of PD‐L1.

## Data Availability

The data that support the findings of this study are available from the corresponding author (joaquim_bellmunt@dfci.harvard.edu) upon reasonable request.
