# Supplementary material for: Enhancing immunotherapy through PD‐L1 upregulation: the promising combination of anti‐PD‐L1 plus mTOR inhibitors
Source: Mol Oncol. 2024 Sep 11;19(1):151–72. doi: 10.1002/1878-0261.13699 (PMC11705730; doi:10.1002/1878-0261.13699)
Supplement: Supplementary file 1 — Fig. S1. Characterization of the expression of PD‐L1 in bladder cancer cells. Fig. S2. Effects of TAK‐228 alone or in combination on PD‐L1 in cells and/or in ex vivo cells derived xenografts. Fig. S3. Mechanism of PD‐L1 regulation by mTOR inhibitors. Fig. S4. Expression of IFNGR1 in bladder cancer cells. Fig. S5. Analysis of the activation of CD8+ T cells in PBMC. Fig. S6. Co‐culture experiments with PBMC and tumor cells. Table S1. Oligonucleotide sequence of primers used for qRT‐PCR. Table S2. Effect of TAK‐228 in patient‐derived explants (PDE) treated ex vivo. Clinical information and results of the IHC staining of PD‐L1. [file MOL2-19-151-s001.zip › MOL2_13699-Legends.docx]

**SUPPORTING INFORMATION**

**Supplementary Figure 1. Characterization of the expression of PD-L1 in bladder cancer cells. A. Characterization of the glycosylation of PD-L1.** Cells were treated with tunicamycin (10 or 20µM) O/N. The graph shows the levels of PD-L1 in each condition, expressed as fold induction versus control arbitrarily set at 1. The graph shows the levels of total PD-L1, glycosylated PD-L1, non-glycosylated PD-L1. Representative blots from 2 independent experiments are shown. Error bands indicate standard deviation (SD). **B and C. Genetic analysis of the status of the PD-L1 gene. B.** Summary of the copy number alterations of *CD274* (PD-L1) and the status of chromosome 9 analyzed by FISH. **C.** Representative images of the FISH analysis in RT4 and HT-1197 cells. Images from representative nuclei are acquired at a magnification of 10*×*100x = 1000x oil objective. Scale bar = 5µm. Images were captured with Cytovision (Leica Biosystems). **D. Analysis of the mutations and amplifications in tumor samples from patients.** In silico analysis of 411 Bladder urothelial carcinoma obtained from the TCGA PanCan 2018 and the MSK Our Urol 2017 public datasets. **E.** **Effect of IFNγ on mRNA expression of PD-L1.** Cells were seeded and after 24 hours the cells were treated with IFNγ (50ng/mL) for 48 hours. PD-L1 mRNA was detected by qRT-PCR. mRNA levels were normalized to the control levels (set at 1). The average of three independent experiments is shown. Error bands indicate standard deviation (SD).

**Supplementary Figure 2. Effects of TAK-228 alone or in combination on PD-L1 in cells and/or in *ex vivo* cells derived xenografts. A. Effect of different doses of TAK-228 on PD-L1 expression in CAL-29 cells.** Cells were treated with increasing doses of TAK-228 (1, 10, 25 and 50nM) for 48 hours. Cellular extracts were analyzed by western blot. The graph shows the levels of PD-L1 in each condition, expressed as fold induction versus control arbitrarily set at 1 from three independent experiments. **B. Effects of TAK-228 in the expression of PD-L1 in the cell surface of CAL-29 cells.** The graph shows the fold-change MFI ratio (mean fluorescence intensity ratio) for PD-L1 relative to control cells. Representative image of the PD-L1 expression detected by flow cytometry from three independent experiments. **C. Effect of TAK-228 in combination with paclitaxel on PD-L1 expression in T24 and CAL-29 cells.** Cells were treated with TAK-228 (50nM) and/or paclitaxel (10nM) for 48 hours. Cellular extracts were analyzed by western blot. The graph shows the levels of PD-L1 in each cell line, expressed as fold induction versus control arbitrarily set at 1 from three independent experiments. Asterisks indicate significant differences between groups by Student t-test (**P*<0.05). **D and E. Effect of TAK-228 in tumor samples from CAL-29 tumor xenografts and T24 tumor xenografts.** Tumors were harvested from mice, fragmented, and treated with TAK-228 (T24: 50nM and CAL-29: 20nM). FFPE blocks were prepared from tumor fragments after 24 hours of treatment with TAK-228. Tumor fragments were stained for PD-L1. Necrotic areas were excluded from the analysis, and only PD-L1 staining (in brown) in live tumor cells was quantified. **D.** Table shows the percentage of the PD-L1 staining in tumor cells of CAL-29 and T24 tumor xenografts. **E.** Representative images of PD-L1 for the indicated experimental conditions on freshly *ex vivo* cultured CAL-29 tumor xenografts. Images were acquired at a magnification of 10*×*40x = 400x. Scale bar (20µm) is shown on the bottom left of both images.

Error bands indicate standard deviation (SD).

**Supplementary Figure 3. Mechanism of PD-L1 regulation by mTOR inhibitors.** Cells were seeded in 100mm^2^ dishes and 24 hours later cells were treated as indicated. **A. Effects of everolimus and TAK-228 on the expression of candidate genes in T24 and CAL-29.** The mRNA levels of HIF1A and IL-6 were analyzed by qRT-PCR after the treatment with everolimus (100nM) and TAK-228 (50nM) for 48 hours. The graphs show the levels of HIF1A and IL-6 expressed as fold induction versus control arbitrarily set at 1. The average of three independent experiments is shown. **B. Effects of IFNγ, IFNβ, EGF and TAK-228 on PD-L1 in CAL-29 cells.** Cells were treated with IFNγ (50ng/mL), IFNβ (2700U), EGF (25ng/mL) and TAK-228 (50nM). PD-L1 expression was analyzed by qRT-PCR or western blot. The graphs show the levels of PD-L1 expressed as fold induction versus control arbitrarily set at 1. The average of three independent experiments is shown.  **C. EGFR expression in bladder cancer cells.** Cells were seeded in 100mm^2^ dishes and left untouched for 48 hours. Cellular extracts were analyzed by western blot. The graph shows the basal levels of EGFR from three independent experiments. **D. Effects of the blockage of EGFR by cetuximab in CAL-29 cells.** Cells were treated with cetuximab (15µg/ml) for 3 hours and then EGF (25ng/mL) was added for 48 hours. The graph shows the fold-change MFI ratio (mean fluorescence intensity ratio) for PD-L1 relative to control cells. Representative image of the PD-L1 expression detected by flow cytometry from three independent experiments.

Error bands indicate standard deviation (SD). Asterisks indicate significant differences between groups by Student t-test (**P*<0.05, ***P*<0.01 and ****P*<0.001).

**Supplementary Figure 4. Expression of IFNGR1 in bladder cancer cells. A. Analysis of the mRNA expression levels in human bladder cancer cells.** Analysis of the mRNA of IFNGR1 and IFNGR2 of the cells from the Cancer Cell line Encyclopedia. Data obtained from cBioPortal. **B. Basal expression of IFNGR1.** Cells were seeded in 100mm^2^ dishes and left untouched for 48 hours. Cellular extracts were analyzed by western blot. Representative images of three independent experiments are shown.

**Supplementary Figure 5. Analysis of the activation of CD8+ T cells in PBMC (peripheral blood mononuclear cells).** Gating strategy to detect the expression of CD69 or PD-1 by flow cytometry on CD8+ T cells at 24 hours after stimulation with Dynabeads human T-Activator CD3/CD28. The results of resting PBMC are shown above, and the results of activated PBMC are shown below. Images represent one of the different stocks used in the paper. The tables show the proportions of CD69 and PD-1 in resting and activated CD8+ T cells in each stock of PBMC. The tables show the data for the different stocks of PBMC that were used for each of the several repetitions.

**Supplementary Figure 6. Co-culture experiments with PBMC (peripheral blood mononuclear cells) and tumor cells. A and B. Effect of PBMC on cell viability in a co-culture system with CAL-29 cells.** Cells were seeded in 24-well plates, and 24 hours later, cells were co-cultured with activated PBMC in 3:1 and 10:1 (effector:target) ratios. After the indicated time, cells were washed with PBS and stained with crystal violet. Graphs show the percentage of cell viability relative to the control cells without PBMC. **A. Effect of different stocks of PBMC on the cell viability.** Cells were treated with three different stocks of PBMC for 48 hours. Each stock of PBMC is represented by a different color. **B. Effect of PBMC treatment at different times on cell viability.** Cells were treated with PBMC for 48 and 72 hours. **C. Effect of TAK-228 on cell viability in the presence of PBMC.** T24 or CAL-29 cells were seeded in 24-well plates and 24 hours later cells were treated with TAK-228 (50nM). After 24 hours, cells were co-cultured with resting or activated PBMC in 5:1 (effector:target) ratio. After 72 hours cells were washed with PBS and stained with crystal violet. Graphs show the percentage of cell viability relative to the cells treated with resting PBMC. % of inhibition in each condition = % of cell viability (resting PBMC) - % of cell viability (activated PBMC), considering 100% the resting PBMC condition in all the conditions. Blue column: control cells (without PBMC), green column: resting PBMC and orange column: activated PBMC. Graphs show the average of two different experiments. **D. Effect of TAK-228 on cell viability in the presence of PBMC using two different treatment schedules.** T24 cells were treated in two ways: sequentially, first with TAK-228 (50nM) for 24 hours, then adding PBMC for 72 hours (left); or concomitantly with TAK-228 (50nM) and PBMC (right) for 72 hours. At the end of the experiment, cell viability was analyzed as indicated in section C in this figure legend. **E. Effect of TAK-228 on the cell viability of PBMC.** PBMC were seeded in a 96-well plate (100,000 cells/well), and 24 hours later, cells were treated with TAK-228 (10, 25, and 50 nM). After 72 hours, cell viability was measured through MTS. The results are expressed as a percentage of viable cells in each condition versus control cells. **F.** **Expression of PD-L1 on the cell surface after the treatment with atezolizumab.** Cells were seeded, and 24 hours later, cells were treated with IFNγ (50ng/mL) for 48 hours. Then, atezolizumab (10 µg/ml) was added, as indicated in the figure, for 3 hours. Then, cells were stained with anti-PD-L1. A representative image of the PD-L1 expression detected by flow cytometry in T24 (left) and CAL-29 (right) is shown. The MFI ratio (mean fluorescence intensity ratio) of each condition versus untreated cells is shown on the right of the image.  **G. Effect of TAK-228 in combination with atezolizumab on cell viability in the presence of activated PBMC.** Cells were seeded in 24-well plates and 24 hours later cells were treated with TAK-228 (50nM). After 48 hours, atezolizumab (10µg/ml) was added. After three hours, cells were co-cultured with activated PBMC in a 10:1 ratio. After 72 hours, the luciferase activity of CAL-29-GFP+-Luc+ cells was analyzed with the luciferase assay system kit. Graphs show the percentage of cell viability relative to the control cells. Color dots represent the replicates of four different experiments.

Error bands indicate standard deviation (SD). Asterisks indicate significant differences between groups by Student t-test (**P*<0.05, ***P*<0.01, ****P*<0.001 and *****P*<0.0001).

**Supplementary Table 1. Oligonucleotide sequence of primers used for qRT-PCR.** Oligonucleotide sequence (5’ to 3’) and annealing temperature of the analyzed genes.

**Supplementary Table 2. Effect of TAK-228 in patient-derived explants (PDE) treated *ex vivo.*  Clinical information and results of the IHC staining of PD-L1.** The first column indicates the patient. The second column shows the clinical information of the tumor samples: grade and stage. Grade: G1: low grade (mild dysplasia), G2: high grade (moderate dysplasia), and G3: high grade (severe dysplasia). Stage: Ta: noninvasive papillary carcinoma and Tis: carcinoma in situ (CIS) or a “flat tumor”. The third column shows the percentage of the tumor cells with low (+), moderate (++) or intense PD-L1 staining (22C3 Intensity) in control and TAK-228-treated samples
