# Supplementary material for: Enhancing immunotherapy through PD‐L1 upregulation: the promising combination of anti‐PD‐L1 plus mTOR inhibitors
Source: Mol Oncol. 2024 Sep 11;19(1):151–72. doi: 10.1002/1878-0261.13699 (PMC11705730; doi:10.1002/1878-0261.13699)
Supplement: Supplementary file 1 — Fig. S1. Characterization of the expression of PD‐L1 in bladder cancer cells. Fig. S2. Effects of TAK‐228 alone or in combination on PD‐L1 in cells and/or in ex vivo cells derived xenografts. Fig. S3. Mechanism of PD‐L1 regulation by mTOR inhibitors. Fig. S4. Expression of IFNGR1 in bladder cancer cells. Fig. S5. Analysis of the activation of CD8+ T cells in PBMC. Fig. S6. Co‐culture experiments with PBMC and tumor cells. Table S1. Oligonucleotide sequence of primers used for qRT‐PCR. Table S2. Effect of TAK‐228 in patient‐derived explants (PDE) treated ex vivo. Clinical information and results of the IHC staining of PD‐L1. [file MOL2-19-151-s001.zip › mol213699-sup-0001-Supinfo.pdf]

SUPPLEMENTARY FIGURE 1

A

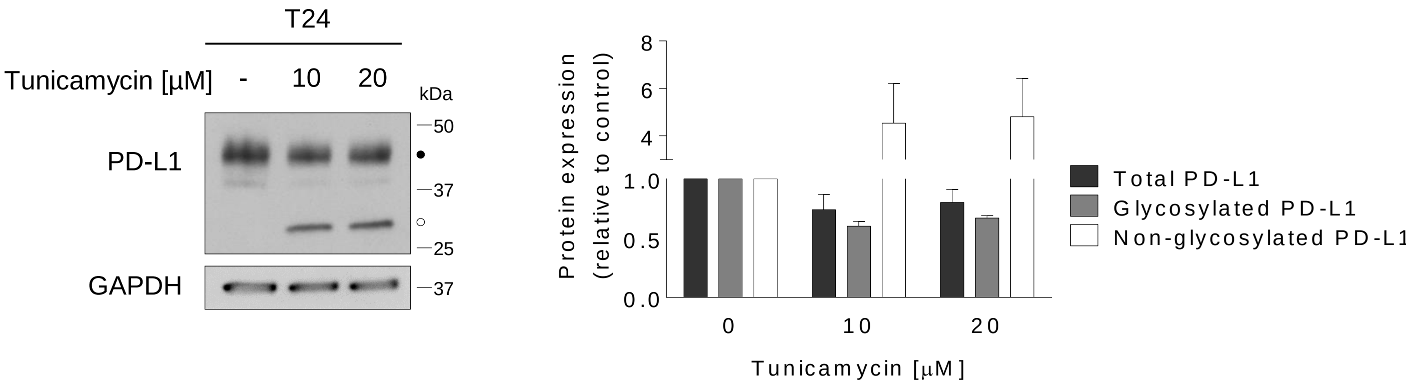

B

Cell line	PDL1-2/CEP9	Status of chromosome 9
T24	4,15 / 2,36 = 1,76	Copy number gain, no amplification*
RT4	2,9 / 2,9 =1	Chromosome 9 trisomy
UM-UC-3	4,8 / 3,8=1,2	Chromosome 9 polysomy (5 copies)
CAL-29	3 / 3 =1	Chromosome 9 trisomy
TCCSUP	3 / 3 = 1	Chromosome 9 trisomy
HT-1197	4,4 / 4,6 = 1	Chromosome 9 polysomy (4-5 copies)
J82	3 / 3 = 1	Chromosome 9 trisomy

*Amplification: ratio ≥ 2

C

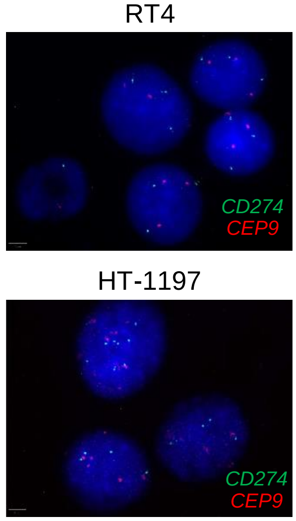

D

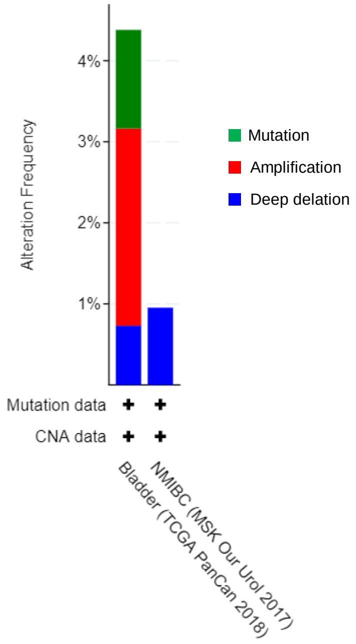

E

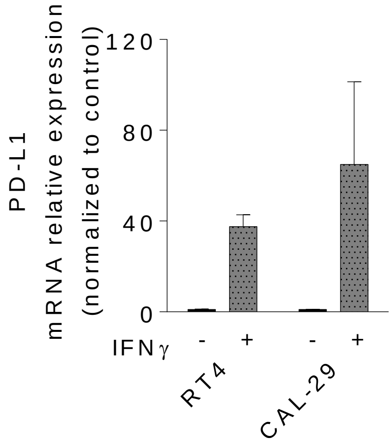

SUPPLEMENTARY FIGURE 2

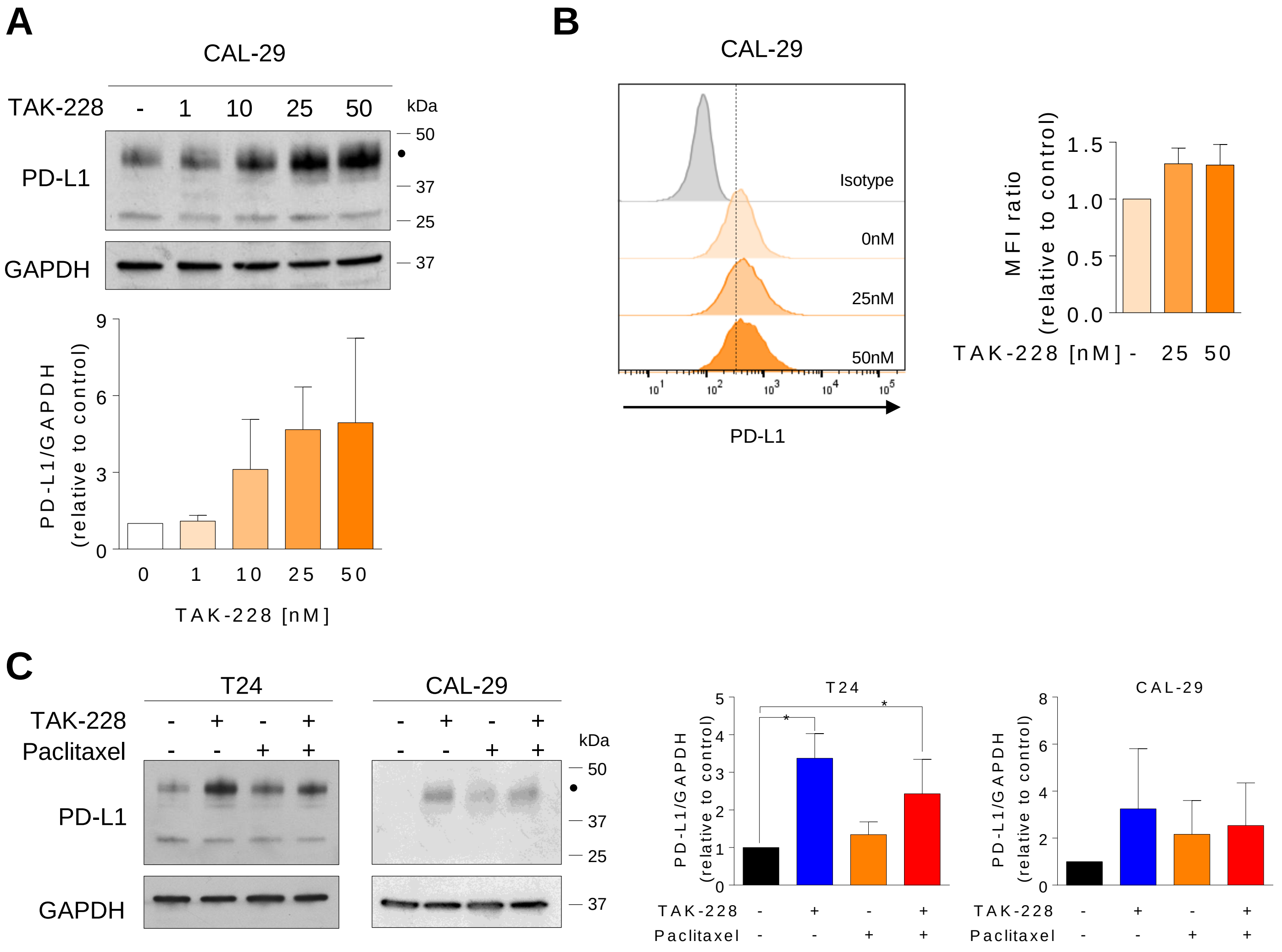

D

3D fragments	PD-L1 staining in tumor cells
CAL-29	Control: 5% TAK-228: 20%
T24	Control: 30% TAK-228: 5%

E

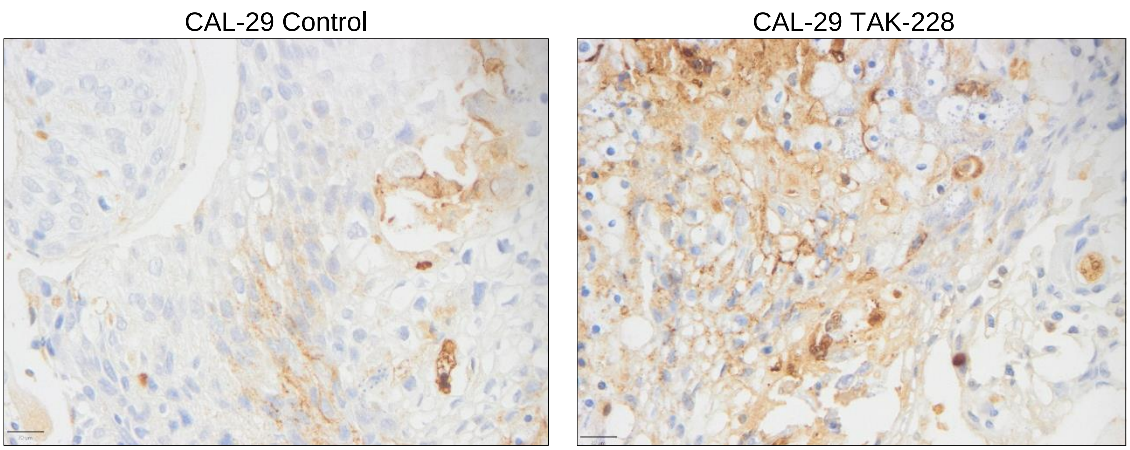

SUPPLEMENTARY FIGURE 3

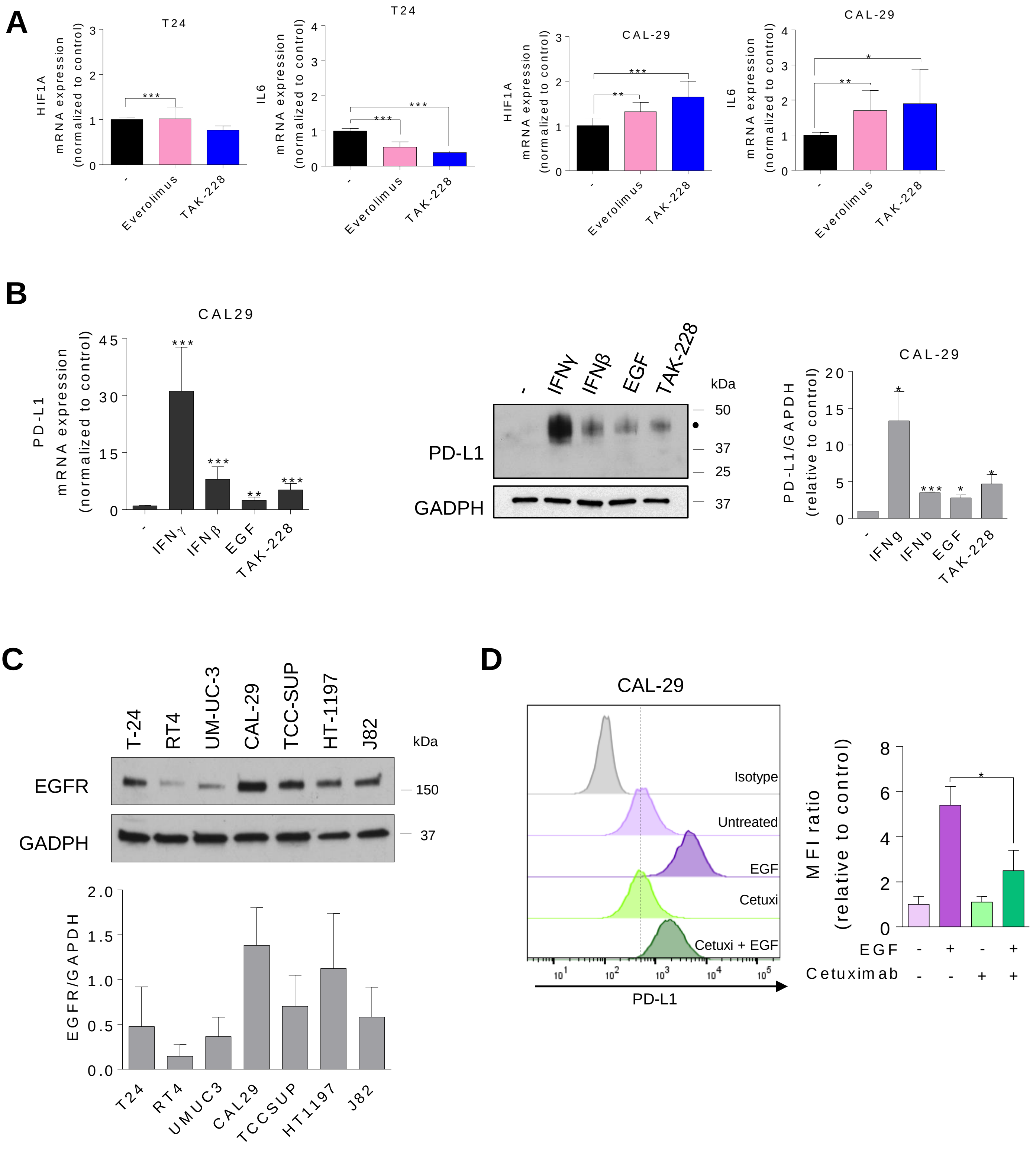

SUPPLEMENTARY FIGURE 4

A

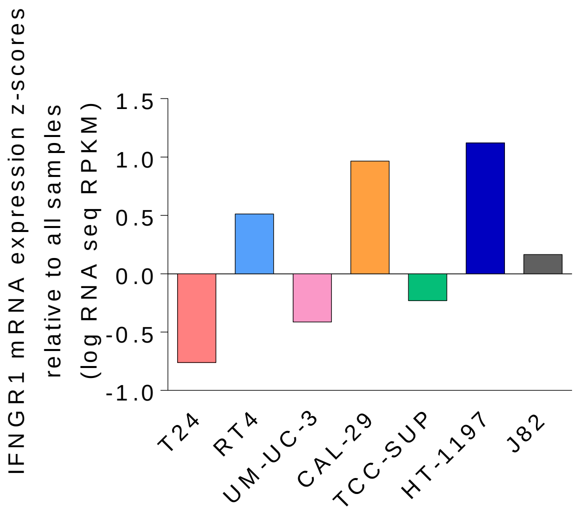

B

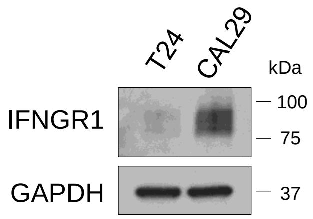

SUPPLEMENTARY FIGURE 5

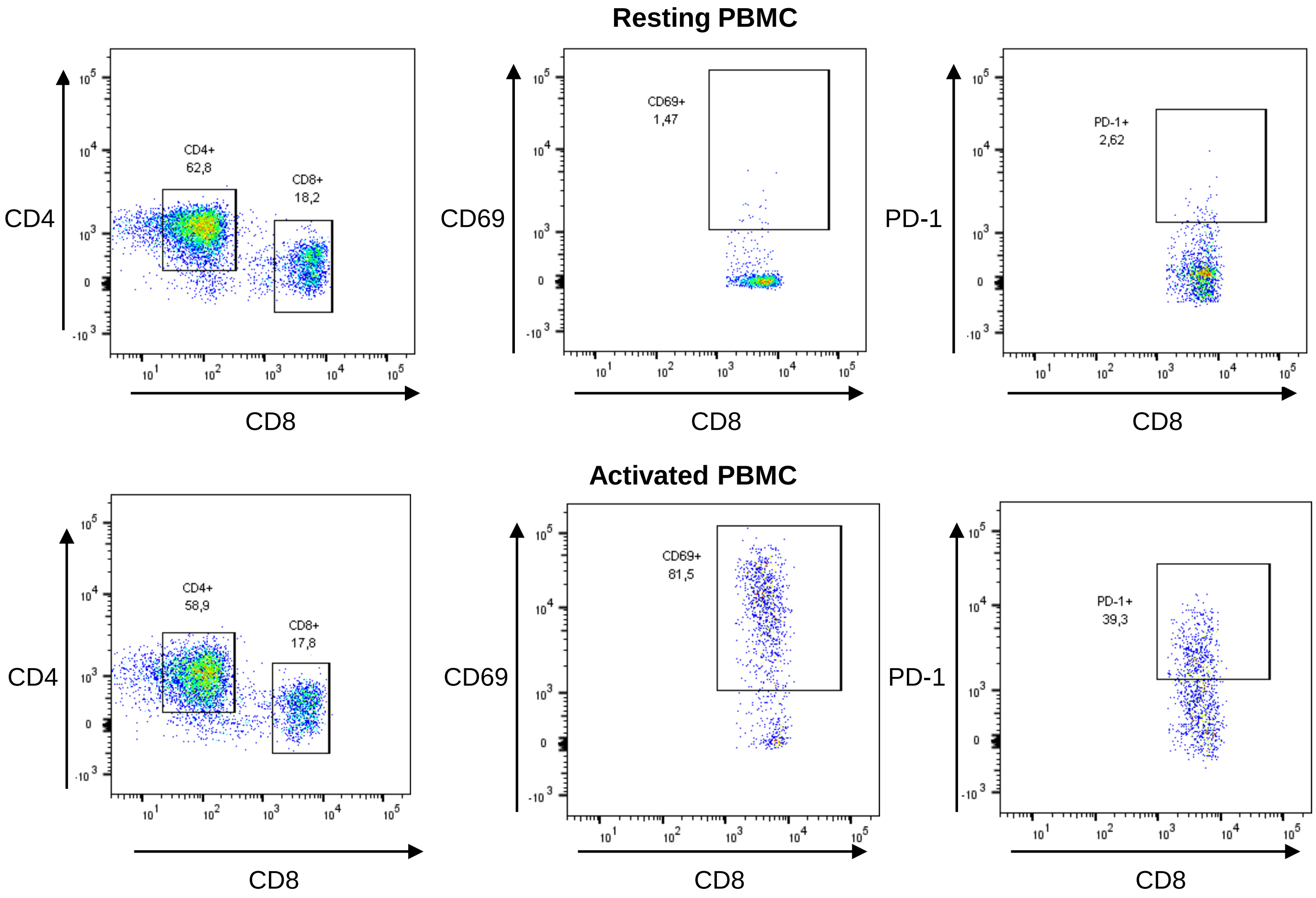

	% CD69	
**PBMC**	**Resting**	**Activated**
Stock 1	1.47	81.5
Stock 1	0.29	70.9
Stock 2	0.32	57.9
Stock 2	0.36	87.4
Stock 3	0.37	69.6
Stock 4	0.17	78.4
	**0.29-1.47**	**57.9 – 81.5**

	% PD-1	
**PBMC**	**Resting**	**Activated**
Stock 1	2.62	39.3
Stock 1	0.22	21.0
Stock 2	0.29	5.13
Stock 2	0.32	29.4
Stock 3	0.18	20.9
Stock 4	1.45	27.3
	**0.18-2.62**	**5.13 – 39.3**

SUPPLEMENTARY FIGURE 6

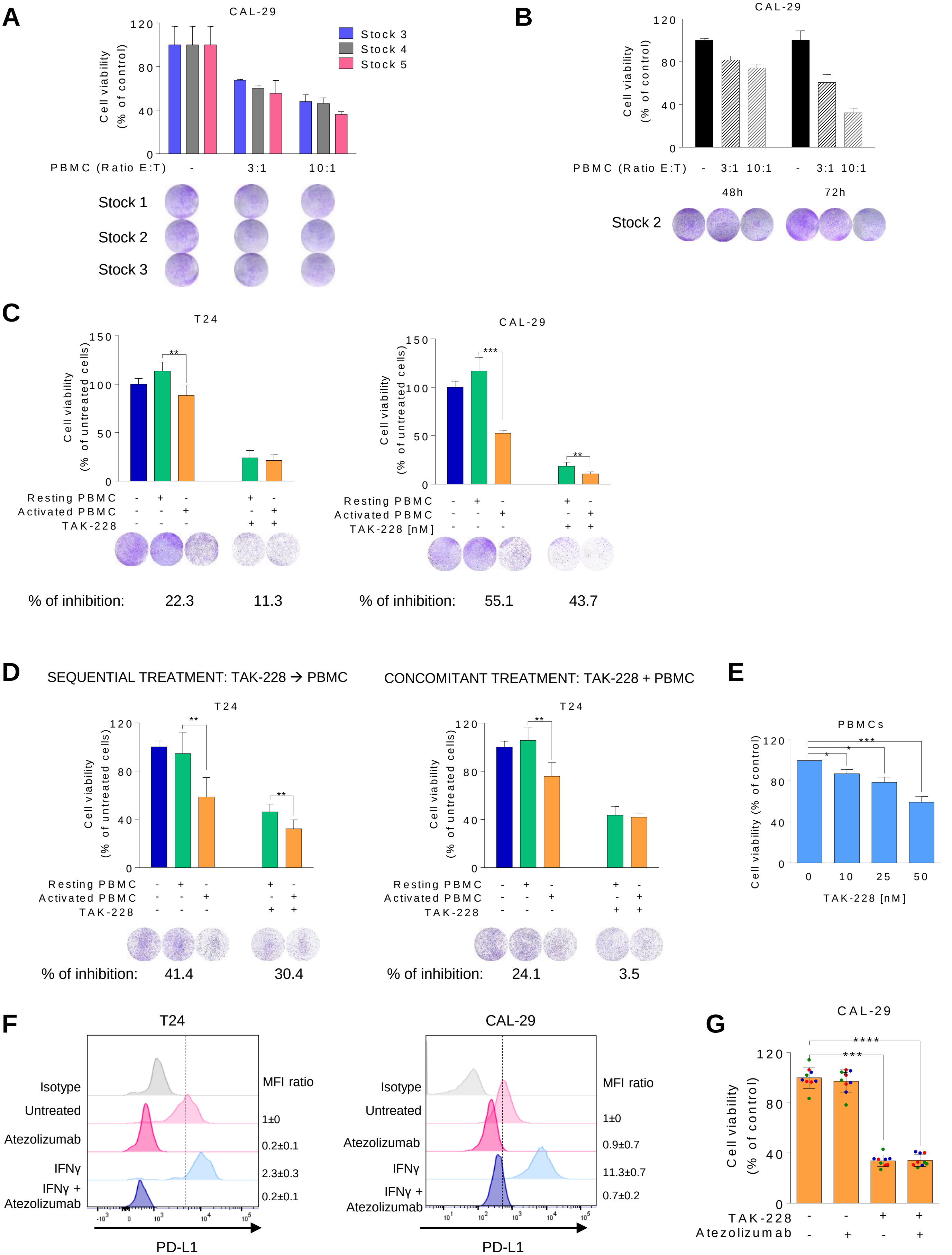

SUPPLEMENTARY TABLE 1

Nucleotide sequence of primers used for qRT-PCR

Protein name	Gene name	Oligonucleotide sequence (5' to 3')	Annealing temperature
PD-L1	*CD274*	**Forward** GTGGCATCCAAGATACAAACTCAA<br>**Reverse** TCCTTCCTCTTGTCACGCTCA	60ºC
IFNβ	*IFNB1*	**Forward** ATGACCAACAAGTGTCTCCTCC<br>**Reverse** GGAATCCAAGCAAGTTGTAGCTC	60ºC
EGF	*EGF*	**Forward** CGTGTCGTGAAGGTTTTATG<br>**Reverse** GTTCTTTAGATCAACTTCACC	60ºC
HIF-1α	*HIF1A*	**Forward** CCACAGGACAGTACAGGATG<br>**Reverse** TCAAGTCGTGCTGAATAATACC	60ºC
IL-6	*IL6*	**Forward** CCGGGAACGAAAGAGAAGCT<br>**Reverse** GCGCTTGTGGAGAAGGAGTT	60ºC
GAPDH	*GAPDH*	**Forward** GGAGTCAACGGATTTGGTCGTA<br>**Reverse** GGCAACAATATCCACTTTACCAGAGT	60ºC
ATP5E	*ATP5F1E*	**Forward** GATCTGGGAGTATCGGATG<br>**Reverse** CCGGCGTCTTGGCGATTC	60ºC

SUPPLEMENTARY TABLE 2

Patient	Grade and stage (GpT)	PD-L1 staining (22C3 Intensity)
1	G1pTa	Control: 0%<br>TAK-228: No viable tumor cells
2	G2pTa	Control: 80%+ 20%++<br>TAK-228: 50%+ 50%++
3	G3pTa	Control: 90%+ 10%++<br>TAK-228: 85%+ 15%++
4	G3pT1a	Control: 0%<br>TAK-228: 0%
5	G3pT1a<br>G3pTis	Control: 85%+ 14%++ 1%+++<br>TAK-228: 80%+ 19%++ 1%+++
